# Plant nitrogen retention in alpine grasslands of the Tibetan Plateau under multi-level nitrogen addition

**DOI:** 10.1038/s41598-023-27392-y

**Published:** 2023-01-17

**Authors:** Jiaoneng Yu, Xu-Ri, Songbo Qu, Fengzi Li, Da Wei, Almaz Borjigidai

**Affiliations:** 1 Key Laboratory of Ethnomedicine, Ministry of Education, Minzu University of China, Beijing, 100081 China; 2 State Key Laboratory of Tibetan Plateau Earth System, Resources and Environment, Institute of Tibetan Plateau Research, Chinese Academy of Sciences, Beijing, 100101 China; 3 Inner Mongolia Academy of Forestry Sciences, Hohhot, 010010 China; 4 Key Laboratory of Mountain Surface Processes and Ecological Regulation, Institute of Mountain Hazards and Environment, Chinese Academy of Sciences, Chengdu, 610041 China

**Keywords:** Element cycles, Climate-change ecology, Community ecology, Grassland ecology

## Abstract

Nitrogen (N) deposition might alleviate degradation of alpine grassland caused by N limitation on the Tibetan Plateau (TP). To determine such limitation and quantify the N-induced N retention in plant, a six-year fertilization experiment with six levels of N addition rates (0, 1, 2, 4, 8 and 16 g N m^−2^ yr^−1^) was conducted in the Namco alpine steppe and additional 89 experiments with multi-level N addition were also synthesized worldwide among which 27 sites were on the TP. In general, N addition promoted N retention in plants, and this increasing trend diminished at the critical N rate (N_cr_). The maximum N retention capacity (MNRC) of plants at N_cr_ was strongly correlated with initial aboveground net primary productivity with a slope of 0.02, and the MNRC of grasslands globally ranged from 0.35 to 42.59 g N m^−2^ yr^−1^, approximately account for 39% of N_cr_. Tibetan alpine grassland had a low average MNRC (2.24 g N m^−2^ yr^−1^) with distinct regional characteristic, which was much lower in the western TP (0.80 g N m^−2^ yr^−1^) than the eastern TP (4.10 g N m^−2^ yr^−1^). Our results inferred 0.33–1.21 Tg N yr^−1^ (0.22–0.79 g N m^−2^ yr^−1^) can be retained and 5.65–20.11 Tg C yr^−1^ (3.67–13.06 g C m^−2^ yr^−1^) can be gained by Tibetan alpine grasslands under current N deposition level. With the aggravation of N deposition, the alpine steppe ecosystem might continuously absorb N and C until N deposition reaches N_cr_.

## Introduction

Nitrogen (N) cycling has been dramatically changed due to anthropogenic activities such as the combustion of fossil fuels and the use of agricultural fertilizers, with N inputs to terrestrial ecosystems doubled in the past century^1,2^. Previous study showed that the recent rate of N deposition has been 3–5 folds higher than the last century^3^. In addition, N deposition over the continental ecosystems would vary between 60 and 100 Tg N yr^−1^ by 2100^4^. Available records of δ^15^N in soil sediments suggest that terrestrial ecosystems can continuously absorb reactive N released by human activities^5^. However, we still do not know how much N can be retained in terrestrial ecosystems and its underlying mechanisms^5^.

Many changes in the structure, function and stability of ecosystem may occur when the N inputs into terrestrial ecosystems increase^6,7^. As a primary nutrient for plant growth in many terrestrial ecosystems^8^, N fertilizers have been widely used to enhance the productivity of farmlands and pasturelands. However, due to the different responses to increasing N among individual species and communities determined by their inherently different N use efficiency and strategies^6^, experiments have indicated that increasing N input can lead to cascading effects such as long-term species composition change and biodiversity loss^9,10^. As a result, a better understanding of biodiversity, species, and community structure is critical for further ecosystem management and protection under the background of increasing N input^8^.

Changes in ecological conditions caused by N deposition may exert an important influence on ecosystem properties and biogeochemical cycling. For instance, N deposition promotes the uptake and assimilation of atmospheric CO_2_ by plants, accelerates the release of greenhouse gases, and affects nitrification and denitrification^11–13^. As defined by nutrient limitation, an ecosystem is limited if N addition leading to the increase of biomass or the rate of biological processes^14^. Therefore, under the circumstance of N limitation, exogenous N input can stimulate plant growth, increase net primary productivity and then promote C and N accumulation^11,15^. On the contrary, when exogenous N exceeds the capacity for plants, soil and microbes to uptake and retention, the negative effects of redundant N are obvious: photosynthesis and productivity decline and then plants stop growing or tend to die^16,17^. The N saturation point is defined as the state of an ecosystem when the supply of reactive N (mainly ammonia and nitrate) in the environment exceeds the demand of plants and soil microorganisms^16,18^, and critical rates are the amount of fertilizer input at this point^19,20^. Assessing the critical N rate (N_cr_) at saturation point is an important goal for land managers^19^.

Although N limitation is a widespread phenomenon in terrestrial ecosystems^11^, the knowledge about limitation of grassland ecosystems is unclear, so a better understanding of N limitation and the maximum N retention and C gain at N_cr_ is essential to our comprehension of ecosystem N and C cycle. Many indicators are used to assess the status of N limitation, such as δ^15^N of plants and soil, foliar N:P ratios and plant N stress index, etc.^14,17^. All of them have advantages and limitations, and cannot evaluate N limitation of exogenous N addition effectively^21^. Fertilization is a key measurement to restore the ecological functions of degraded grasslands^22^. While previous work has often focused on the response of aboveground net primary productivity (ANPP) to N addition and discussed the issue of ecosystem N saturation threshold^17,23,24^, little attention has been paid to the response of plant N retention to N addition. Therefore, fertilization experiments are still widely regarded as a method to assess N limitation through observing whether plants NPP increase and how much C and N can be accumulated in the ecosystem^11,21^.

**Figure 1:**
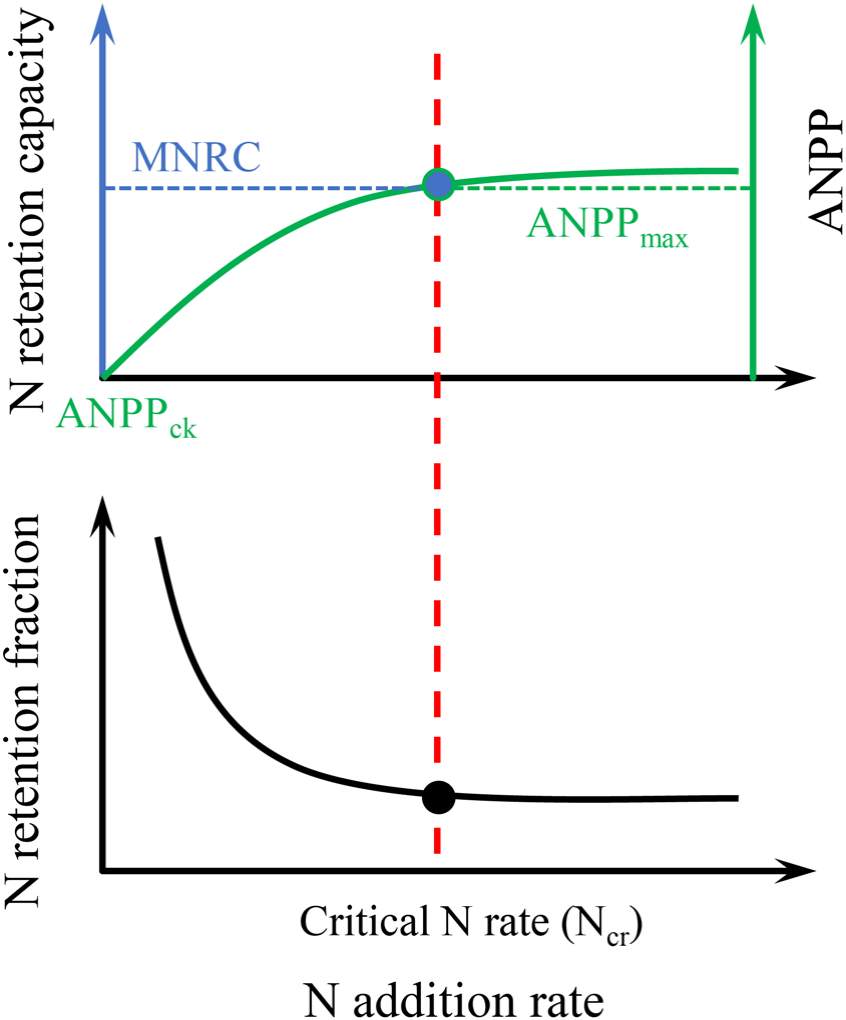
Patterns of plant aboveground net primary productivity (ANPP), N retention capacity and N retention fraction (NRF) response to N addition. ANPP_ck_ is the initial ANPP without exogenous N addition. ANPP_max_ is the saturated ANPP at critical N rate (N_cr_). MNRC is the maximum N retention capacity at N_cr_.

Known as the third pole of the earth, the Tibetan Plateau (TP) covers an area of about 2.57 million km^2^ with more than 60% (~ 1.54 million km^2^) covered by alpine grassland^25^ and most grassland is above 4000 m above sea level^26^. However, due to the particularity of its spatial characteristics, Tibetan alpine ecosystems are much more sensitive to climate change and are known as the “pre-warning region”, playing a vital role in not just Asia but also in global climate behavior^27^. Under the influence of climate change and long-term grazing, TP alpine grasslands are facing degradation^28,29^. The plant and soil N isotope measurement indicated that N limitation in this region might be aggravated^30^. The TP is now experiencing an intensely increase in N deposition rates with the range from 0.32 to 1.23 g N m^−2^ yr^−1^^31,32^ and N fertilization has also been applied to restore degraded grasslands and promote grass productivity for grazing^33–35^. These situations significantly affect the biochemical processes of Tibetan alpine ecosystems^36^. Given the uncertainty of future N deposition, we do not know how much exogenous N can be retained by plants in alpine grasslands and how much N input (N_cr_) is required to reach the saturation point (Fig. 1).

We assumed the greater N limitation, the greater retention of external N in the plant. To understand the impact of elevating N deposition on alpine grasslands community composition and quantify N limitation, we conducted a 6-year field experiment with six levels of N addition (0, 1, 2, 4, 8 and 16 g N m^−2^ yr^−1^) at an alpine steppe site on the TP. We also synthesized 89 (Appendix S2: Fig. S1) experiments with at least four N input levels in worldwide grasslands for comparison, in which 27 sites were located on the TP. The aim of this study was to quantify how much exogenous N can be retained in the plant pool of alpine grassland.

## Methods

### Study site

The field experiment was conducted at Namco Station (30°47’N, 90°58’E, altitude 4730 m) of the Institute of Tibetan Plateau Research, Chinese Academy of Sciences (ITPCAS), which is located in the alpine steppes of TP in China. The experiment was permitted by ITPCAS, complied with local and national guidelines and regulations. From 2006 to 2017, the mean annual temperature (MAT) and mean annual precipitation (MAP) was about − 0.6 °C and 406 mm, respectively. Monthly mean temperature varied from − 10.8 °C in January to 9.1 °C in July and most of the precipitation occurred from May to October^37,38^. During our six-year observations (2010, 2011, 2012, 2013, 2015 and 2017), climate change during the growing season from May to September varied differently, with the annual precipitation ranged from 255.9 mm to 493.8 mm and the MAT from 6.7 to 7.4 °C. *Androsace tapete, Kobresia pygmaea, Stipa purpurea* and *Leontopodium pusillum* were the dominant plant species at the alpine steppe.

### Experimental design and treatments

The long-term experiment began in May, 2010. Three homogenous plots were randomly arranged as replicates at the alpine steppe and six subplots (~ 13 m^2^) were distributed in each plot by a cycle, with a 2 m buffer zone between each adjacent subplot (Appendix S1: Fig. S1). In this experiment, six treatments of N fertilization rate (0, 1, 2, 4, 8, and 16 g N m^−2^ yr^−1^) were clockwise applied in each subplot. The subplots of 0 g N m^−2^ yr^−1^ were control group. We sprayed NH_4_NO_3_ solution on the first day of each month in the growing season (from May to September) each year. After fertilizing, we rinsed plant residual fertilizer with a little deionized water (no more than 2 mm rainfall). For the control groups, we added equivalent amount of water. The experiment was conducted from 2010 to 2017 (it should be pointed out that there was no fertilization in 2014 and 2016).

### Sampling and measurements

The samples were collected with the training and permission of ITPCAS and involved plants that are common species and not endangered or protected. The identification of the plants was done by referring to a book of Chen and Yang^39^. Pictures of the corresponding specimens can be seen on the website of ITPCAS (http://itpcas.cas.cn/kxcb/kxtp/nmc_normal_plant/).

Vegetation samples were collected in August in 2011 and repeated at the same time in 2012, 2013, 2015 and 2017. We established one 50 × 50 cm quadrat in each subplot, clipped aboveground biomass (AGB) and sorted species by families. The biomass was used to measure ANPP (g m^−2^ yr^−1^). Following aboveground portion collected, we used three soil cores (5 cm diameter) to collect the belowground roots at 0–30 cm depth and mixed into one sample, which were used to assess belowground net primary productivity (BNPP, g m^−2^ yr^−1^). The roots were cleaned with running water to remove sand and stones.

Both plant and root samples were dried at 75 °C for 48 h and then ground into powder (particle size ~ 5 μm) by a laboratory mixer mill (MM400, Retsch). To determine N and C content of plants, we weighed the samples into tin capsules and measured with the elemental analyzer (MAT253, Finnigan MAT GmbH, Germany).

### Estimation of the critical N rate (N_cr_), N retention fraction (NRF), N retention capacity and N-induced C gain

According to the N saturation hypothesis, plant productivity increases gradually during N addition, reaches a maximum at the N_cr_, and eventually declines^16,17^. We considered the N_cr_ to be the rate where ANPP no longer remarkably changed with N addition (Fig. 1).

We defined plant N retention fraction (NRF, %; Eq. 1) as the aboveground N storage caused by unit N addition rate, and N retention capacity (g N m^−2^ yr^−1^; Eq. 2) was the increment of N storage due to exogenous N addition compared to the control^40^. The equations are as following:

1 $$N\;retention\;fraction = \frac{{ANPP_{tr} \times N\;content_{tr} - ANPP_{ck} \times N\;content_{ck} }}{N\;rate}$$

2 $$N\;retention\;capacity = ANPP_{tr} \times N\;content_{tr} - ANPP_{ck} \times N\;content_{ck}$$

where ANPP_tr_ and N content_tr_ (%) refer to those in the treatment (tr) groups, and ANPP_ck_ and N content_ck_ refer to those in the control (ck) groups. These expressions are also used in the following equations (Eqs. 3–5).

The N-induced C gain (g C m^−2^ yr^−1^; Eq. 3) was estimated by the increment of C storage owing to exogenous N addition compared to the control^40^. Maximum N retention capacity (MNRC, Eq. 4) and maximum N-induced C gain (Eq. 5) mean the maximum N and C storage increment in plant caused by exogenous N input at N_cr_, respectively. The formulas are as following:

3 $$N{\text{-}}induced\;C\;gain = ANPP_{tr} \times C\;content_{tr} - ANPP_{ck} \times C\;content_{ck}$$

4 $$MNRC = ANPP_{\max } \times N\;content_{\max } - ANPP_{ck} \times N\;content_{ck}$$

5 $$Maximum\;N{\text{-}}induced\;C\;gain = ANPP_{\max } \times C\;content_{\max } - ANPP_{ck} \times C\;content_{ck}$$

where ANPP_max_, N content_max_ and C content_max_ refer to the value of ANPP, N content and C content at N_cr_, respectively.

### Data synthesis

To evaluate N limitation and saturation on the TP more accurately, we searched papers from the Web of Science (https://www.webofscience.com) and the China National Knowledge Infrastructure (https://www.cnki.net). The keywords used by article searching were: (a) N addition, N deposition or N fertilization, (b) grassland, steppe or meadow. Article selection was based on the following conditions. First, the experimental site must be conducted in a grassland ecosystem. Second, the experiment had at least three N addition levels and a control group. Third, if the experiment lasted for many years, we analyzed data with multi-year average. Based on the above, we collected 89 independent experimental cases. Among these, 27 cases were located on the TP alpine grasslands, 25 in the Inner Mongolia (IM) grasslands and 37 in other terrestrial grasslands (detailed information sees Appendix S2: Table S1).

We extracted ANPP data and N addition rate of these cases and estimated N_cr_ and ANPP_max_ (Appendix S2: Fig. S2). We then calculated NRF, N retention and C gain of each group of data for further analysis (Appendix S2: Table S2). Most of the 89 cases did not have data on N and C content. To facilitate the calculation, we summarized N and C content from 40 articles in the neighboring areas of the cases and divided the N and C content into seven intervals according to the N addition rate (Appendix S2: Table S3 and Fig. S3). The unit of N addition rate was unified to “g N m^−2^ yr^−1^”. All the original data were obtained directly from texts and tables of published papers. If the data were displayed only in graphs, Getdata 2.20 was used to digitize the numerical data. For the estimation of N retention and C gain of the TP at current N deposition rates and future at N_cr_, we fitted the exponential relationship to the data from 27 cases on the TP, and then substituted N rates into the fitted equations (Eq. 6):

6 $$y = a \times \left[ {1 - \exp \left( { - bx} \right)} \right].$$

We also included MAT, MAP, soil C:N ratio, fencing management (fencing or grazing) and grassland type (meadow, steppe and desert steppe) of the experiment sites for exploring the drivers affecting N limitation (Appendix S2: Table S1). When climatic data were missing from the article, MAT and MAP were obtained from the WorldClim (http://www.worldclim.org).

Species were usually divided into four functional groups (grasses, sedges, legumes and forbs) to study the response of species composition to N addition in previous study^41^. We synthesized 13 TP experimental cases (including our field experiment) from the data synthesis and each case included at least three functional groups (detailed references see Appendix S2).

### Statistical analysis

There were 42 species in our field experiment. We divided them by family into eleven groups: *Asteraceae* (forbs), *Poaceae* (grasses), *Leguminosae* (legumes), *Rosaceae* (forbs), *Boraginaceae* (forbs), *Caryophyllaceae* (forbs), *Cyperaceae* (sedges), *Labiatae* (forbs), *Primulaceae* (forbs), *Scrophulariaceae* (forbs) and Others*.* Due to species in the group of Others contributed only 1.22% of AGB, we analyzed AGB and foliar stoichiometry among other ten families (Appendix S1: Table S1). In Namco steppe, forbs, grasses, sedges and legumes accounted for 78.0%, 7.4%, 8.2% and 5.2% of the AGB respectively (Appendix S1: Table S1 and Fig. S2). Such a large number of forbs suggested that our experiment was conducted on a severely degraded grassland.

For our field data, two-way ANOVAs were used to analyze the effects of year, N fertilization rate and their interactions on species AGB. One-way ANOVAs were used to test the response of ANPP, BNPP, root:shoot ratio, species foliar C content, N content and C:N ratio to N addition rate. Duncan’s new multiple range test was used to compare the fertilization influences at each rate in these ANOVAs. Prior to the above ANOVAs, we performed homogeneity of variance test and transformed the data logarithmically when necessary. Simple regression was used to estimate the relevance among ANPP, NRF, N retention capacity and C gain with N addition rates.

Structural equation modeling (SEM) was used to explore complex relationships among multiple variables. To quantify the contribution of drivers such as climate and soil to N_cr_, ANPP, NRF and MNRC, we constructed SEM based on existing ecological knowledge and the possible relationships between variables. We considered environmental factors (MAT, MAP and soil C:N) and ANPP_ck_ as explanatory variables, and N_cr_, NRF and MNRC as response variables. We included the ANPP_ck_ in the SEM rather than the ANPP_max_ because we wonder whether there was a relationship between ANPP in the absence of exogenous N input and the ecosystem N retention in the presence of N saturation. This has important implications for assessing N input. Before constructing the SEM, we excluded collinearity between the factors. In addition, Student’s t-test and one-way ANOVAs were performed to explain the effect of fencing management and grassland type on above response variables, respectively. The SEM was constructed using the R package “piecewiseSEM”^42^. Fisher’s *C* was used to assess the goodness-of-model fit, and AIC was for model comparison.

Given the influence of extreme values in the data synthesis, we calculated the geometric mean of N_cr_, NRF, N retention and N-induced C gain. All statistical analyses were performed with SPSS 26.0 and RStudio (Version 1.2.1335) based on R version 3.6.2 (R Core Team, 2019).

## Results

### Species composition and foliar C:N ratio response to N addition

**Figure 2:**
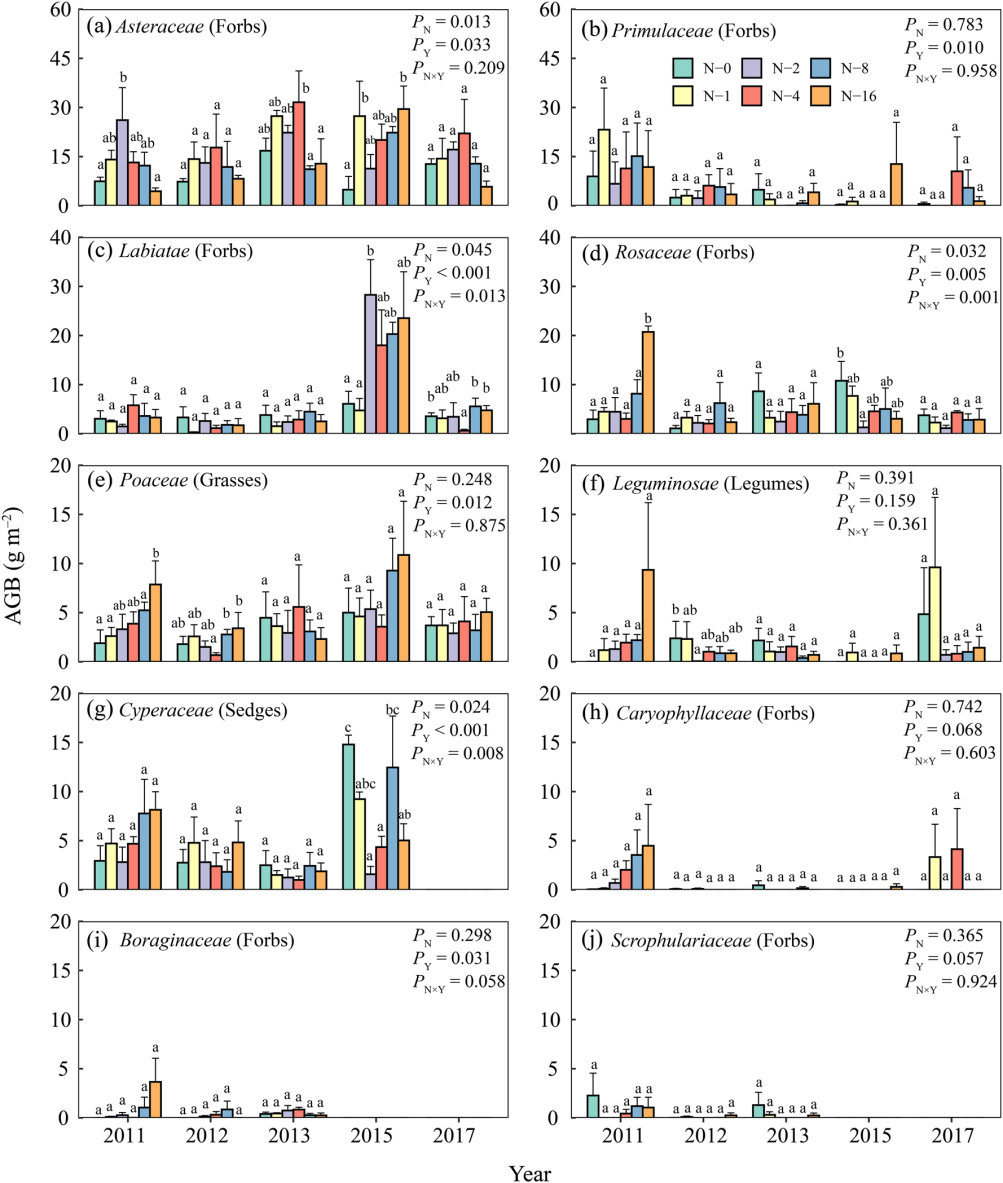
Aboveground biomass (AGB) of species from ten families in response to long term N addition rate. Biomass was collected from 2011 to 2013, 2015, and 2017 in Namco. Data are shown as mean ± SE (*n* = 3). Bars with different letters indicate significant differences (*P* < 0.05) by Duncan’s new multiple range test among fertilization levels. Full statistical results for two-way ANOVA (N, N fertilization; Y, year) are presented in Appendix S1: Table S2.

In our field experiment, the response of biomass to N addition differed between all the ten families, and these differences varied greatly across years (Appendix S1: Table S2). *Asteraceae* (forbs) occupied the largest proportion of biomass (28.89%). AGB of it typically increased with N addition and then decreased, reaching its maximum at the N_cr_ of 4 g N m^−2^ yr^−1^ (Fig. 2a).

The proportion of some forbs disappeared with some grasses increased with N addition. *Primulaceae* (forbs) in some treatment groups began to vanish since 2013 (Fig. 2b). *Rosaceae* (forbs) significantly increased in 2011 and decreased in 2015 (Fig. 2d). *Caryophyllaceae* (forbs), *Boraginaceae* (forbs) and *Scrophulariaceae* (forbs) had all been nearly absent since 2012 (Fig. 2h-j). *Poaceae* (grasses) increased with N addition and no clear N_cr_ was found even under the maximum N fertilization level in 2011 (Fig. 2e). For *Cyperaceae* (sedges) and *Leguminosae* (legumes), we only observed an upward trend in 2011, but they showed little change or even a downward trend in the following years (Fig. 2f,g).

N addition significantly influenced foliar stoichiometry (Appendix S1: Table S3). Foliar N content was enhanced significantly with N addition (*F*_5,138_ = 9.07, *P* < 0.001, Appendix S1: Fig. S3b) while C content did not change obviously (*F*_5,138_ = 0.80, *P* = 0.551; Appendix S1: Fig. S3a). As to species C:N ratios, high N addition led to lower C:N ratio (*F*_5,138_ = 7.78, *P* < 0.001; Appendix S1: Fig. S3c), particularly for *Cyperaceae*, *Asteraceae*, *Rosaceae* and *Labiatae*, while *Leguminosae* remained stable. Moreover, C:N ratio of *Primulaceae* was the largest in our experiment (Appendix S1: Fig. S3c).

### ANPP, BNPP and the root:shoot ratio response to N addition

**Figure 3:**
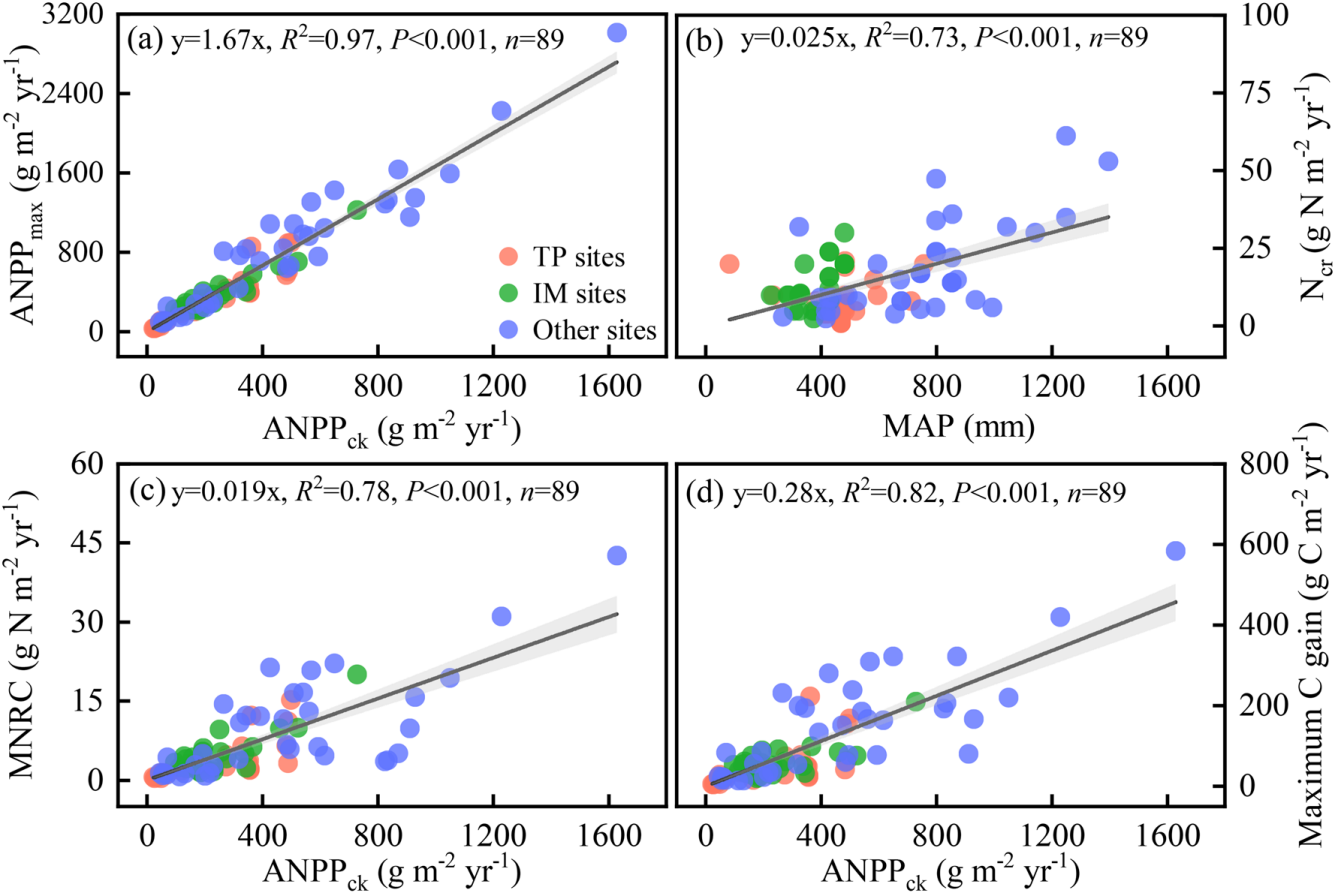
The relationship between the (**a**) maximum aboveground net primary productivity (ANPP_max_) and initial aboveground net primary productivity (ANPP_ck_), (**b**) critical N rate (N_cr_) and mean annual precipitation (MAP), (**c**) maximum N retention capacity (MNRC) and ANPP_ck_, and (**d**) maximum N-induced C gain and ANPP_ck_. The red points represent Tibetan Plateau (TP) sampling plots, green points represent Inner Mongolia (IM) sampling plots, and blue points represent other global grassland sampling plots. Simple regression analyses (Pearson) were implemented. Full regression results are shown in Appendix S2: Table S4. The gray areas indicate the 95% confidence interval.

ANPP showed an increasing trend under the influence of N addition during the field experiment, with significant difference between 16 g N m^−2^ yr^−1^ N addition and the control in 2011 and 2015 (Appendix S1: Fig. S4a). Compared to the control, N addition clearly enhanced ANPP by 158.7% (29.8–77.1 g m^−2^ yr^−1^) and 105.5% (41.8–85.9 g m^−2^ yr^−1^) in 2011 and 2015, respectively. The trend that ANPP increased with N addition was consistent with the results demonstrated in the data synthesis which exhibited that ANPP saturated at N_cr_ in most grasslands (Appendix S2: Fig. S2). We also found a significant linear correlation between ANPP_max_ and initial ANPP (ANPP_ck_). The ANPP_max_ was about 1.67 times higher than that in ANPP_ck_ (*R*^2^ = 0.97, *F*_1,88_ = 2427.58, *P* < 0.001; Fig. 3a and Appendix S2: Table S4). The BNPP had no significant change in response to N addition compared to ANPP, the ratio of root to shoot showed a reduce trend in 2011 and 2015 but an increased trend in 2013 and 2017 (Appendix S1: Fig. S4).

### Spatial variations of NRF

For Namco alpine steppe (our study site), the maximum NRF was 37.16 ± 6.21% at the level of 1 g N m^−2^ yr^−1^, and then decreased with N addition rate until stabilized at 15.75 ± 5.29% at the N_cr_ of 4 g N m^−2^ yr^−1^ (Appendix S1: Fig. S5).

At a larger scale of TP alpine grassland and IM grassland, the NRF showed a trend of decreasing before reaching N_cr_ (Appendix S2: Fig. S4c,d). NRF at N_cr_ was generally higher in the eastern TP (e.g., Haibei, Maqu and Lanzhou) of the alpine grasslands than in the western TP (e.g., Damxung, Gerze, Nagqu, Namco and Nyima) (*t* = 4.85, *df* = 25, *P* < 0.001, Appendix S2: Fig. S4c). Grassland in the eastern TP retained nearly 76.08% of the N (NRF range: 13.30–313.86%) and the western TP retained nearly 13.91% (NRF range: 3.18–36.88%; Table 1). In addition, NRF at N_cr_ in IM (mean: 34.91%) was similar to that in the whole TP (mean: 40.54%; Appendix S2: Fig. S4c,d).

**Table 1:** Global differences in critical N rate (N_cr_), N retention fraction (NRF) at N_cr_, maximum N retention capacity (MNRC) and maximum N-induced C gain.

Region	N_cr_ (g N m^−2^ yr^−1^)	NRF (%)	MNRC (g N m^−2^ yr^−1^)	Maximum N-induced C gain (g C m^−2^ yr^−1^)
Eastern TP, CN	5.39 ± 2.89 c	76.08 ± 2.38 a	4.10 ± 1.90 c	53.27 ± 2.06 b
Western TP, CN	5.77 ± 1.78 c	13.91 ± 2.46 c	0.80 ± 1.74 d	12.73 ± 2.08 c
Inner Mongolia, CN	11.24 ± 1.86 bc	34.91 ± 1.70 abc	3.92 ± 1.91 c	51.49 ± 1.69 b
Eastern USA	46.32 ± 1.49 a	41.38 ± 1.21 ab	19.17 ± 1.23 a	278.20 ± 1.24 a
Middle USA	11.54 ± 1.70 bc	50.71 ± 1.90 a	5.85 ± 2.22 bc	124.89 ± 2.35 ab
Wageningen, NL	31.04 ± 1.39 ab	47.88 ± 1.16 a	14.86 ± 1.43 a	180.82 ± 1.77 a
Eastern AU	8.00 ± 1.00 c	15.64 ± 1.13 bc	1.25 ± 1.13 d	20.80 ± 1.23 c
Western AU	32.00 ± 1.00 ab	38.36 ± 1.09 ab	12.28 ± 1.09 ab	158.54 ± 1.07 a
**Global**	9.79 ± 2.40	39.30 ± 2.34	3.85 ± 2.85	56.93 ± 2.80
**Significance**	*F*_7,66_ = 6.38	*F*_7,66_ = 6.83	*F*_7,66_ = 16.34	*F*_7,66_ = 15.03
	*P* < 0.001	*P* < 0.001	*P* < 0.001	*P* < 0.001

Data are shown as mean ± SD. *F* statistics for one-way ANOVAs are provided. Bold values indicate significance (*P* < 0.05). The same letters in a column indicate insignificant differences (*P* > 0.05) by Duncan’s new multiple range test among regions.

CN, China; USA, America; NL, Netherlands; AU, Australia.

NRF at N_cr_ varied considerably across the global grasslands, with an average NRF of 39.30% at an average N_cr_ of 9.79 g N m^−2^ yr^−1^ (*F*_7,66_ = 6.83, *P* < 0.001; Table 1). In the SEM, factors explained 49% of variance in NRF (Appendix S2: Fig. S5). Among them, initial ANPP and soil C:N were the strongest drivers of NRF, and MAT had a direct positive effect on NRF. In the simple regressions, NRF exhibited a positive linear relationship with initial ANPP (*R*^2^ = 0.54, *F*_1,88_ = 103.81, *P* < 0.001) and a negative non-linear relationship with soil C:N (*R*^2^ = 0.40, *F*_2,78_ = 26.21, *P* < 0.001) (Appendix S2: Table S4 and Fig. S6a,b).

### Spatial variations of MNRC

**Figure 4:**
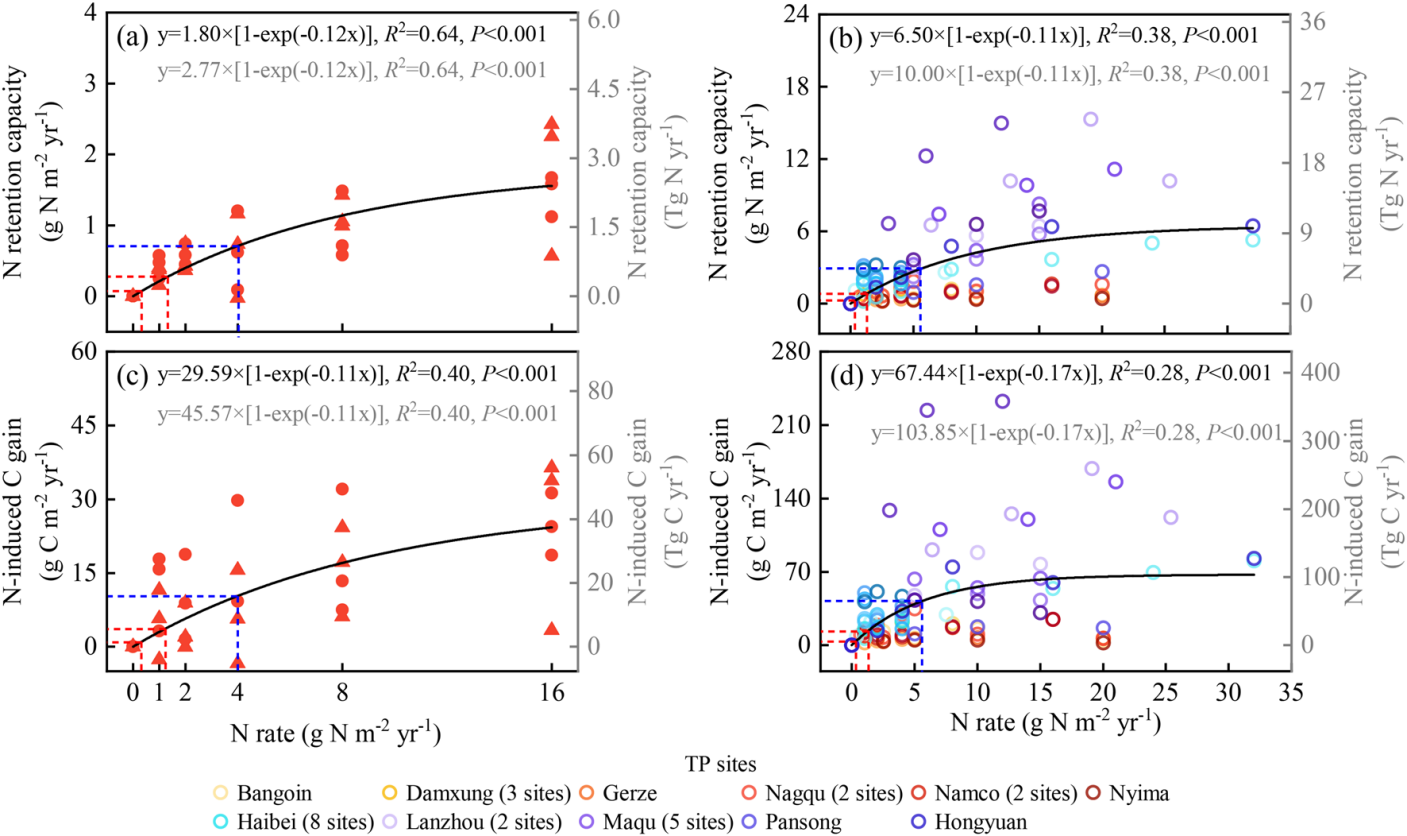
Effects of N addition on N retention capacity and N-induced C gain. (**a**, **c**) N retention and C gain of Namco field experiment in 2011 (circle points) and 2015 (triangle points). (**b**, **d**) N retention and C gain of Tibetan Plateau (TP) experiments in the data synthesis. The left axes indicate the annual N retention and C gain per unit area of the TP. The right axes indicate the total N retention and C gain of the TP per year (based on 1.54 million km^2^ of grassland on the TP). Single regression analyses were implemented. Full regression results are shown in Appendix S2: Table S7. The red dashed line indicates N retention and C gain of Namco and the entire TP grassland at the current rate of N deposition (0.32–1.23 g N m^−2^ yr^−1^). The blue dashed line indicates N retention and C gain under the future N saturation rate (at the N_cr_ of 5.53 g N m^−2^ yr^−1^).

In Namco steppe, N retention rapidly increased to 0.63 ± 0.21 g N m^−2^ yr^−1^ at the N_cr_ of 4 g N m^−2^ yr^−1^, after which this increasing trend became slower (Fig. 4a). Like ANPP, the N retention of plant exhibited a positive nonlinear response with N addition until the input N exceeded plant demand (Appendix S2: Fig. S7).

**Figure 5:**
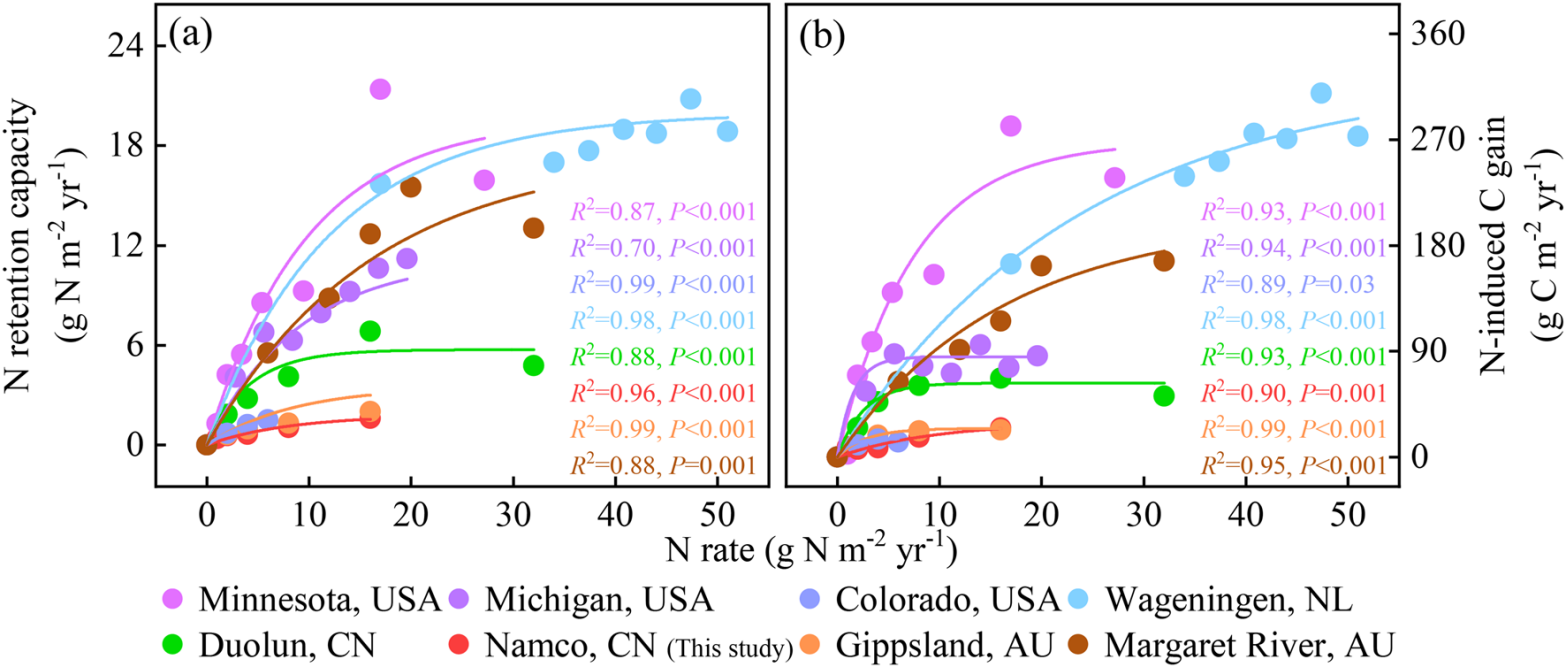
Eight examples showing the response of (**a**) N retention capacity and (**b**) N-induced C gain to N addition rate. USA, America; NL, Netherlands; CN, China; AU, Australia. Each colored line indicates the regression line fitted with the exponential function. Full regression results are shown in Appendix S2: Table S8.

**Figure 6:**
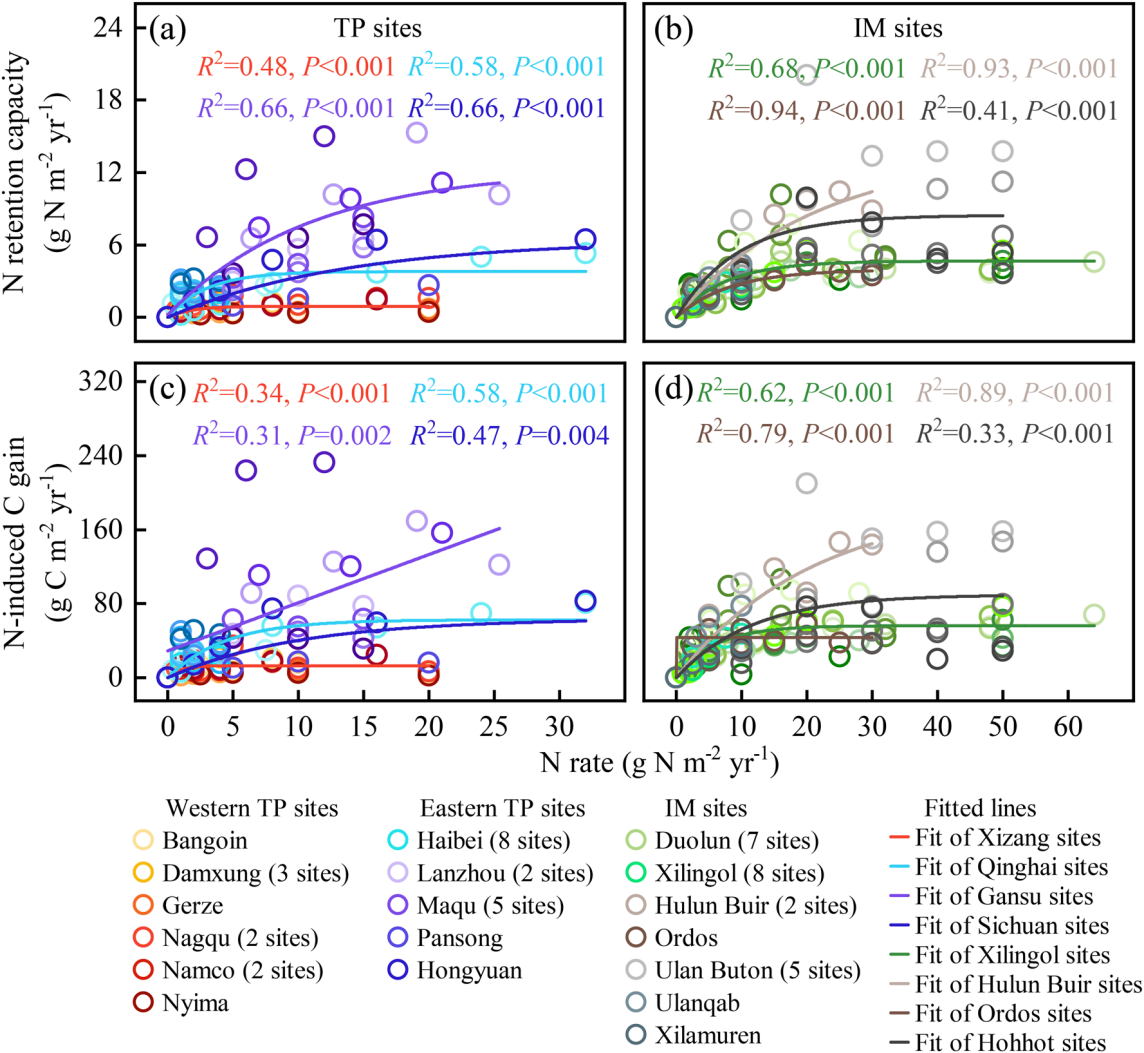
The response of N retention capacity and N-induced C gain in Tibetan Plateau (TP) alpine grassland and Inner Mongolia (IM) grassland. The left panels represent TP sample plots and the right panels represent IM sample plots. Single regression analyses were implemented separately for the western TP (Xizang, *n* = 49), eastern TP (Qinghai, *n* = 36; Gansu, *n* = 29; and Sichuan, *n* = 10) and four regions in IM (Xilingol, *n* = 95; Hulun Buir, *n* = 11; Ordos, *n* = 7; and Hohhot, *n* = 30). Full regression results are shown in Appendix S2: Table S9. Points of each color represent a sampling plot and fitting lines of each color represent a region.

Notably, the MNRC in Namco steppe was much lower than that of grasslands elsewhere in the world (Fig. 5a). We also observed that MNRC of eastern TP (e.g., Haibei, Maqu, and Lanzhou; mean: 4.10 g N m^−2^ yr^−1^) at elevations of 3000–3500 m was generally higher than that of the western TP (e.g., Bangoin, Damxung, Gerze, Nagqu, Namco and Nyima; mean: 0.80 g N m^−2^ yr^−1^) at elevations greater than 4000 m (Fig. 6a), and IM had a similar MNRC (mean: 3.92 g N m^−2^ yr^−1^; Fig. 6b) as the eastern TP (e.g., Haibei, Maqu and Lanzhou).

For global grasslands, under elevated N addition, the MNRC in aboveground biomass ranged from 0.35 to 42.59 g N m^−2^ yr^−1^, with an average of 3.85 g N m^−2^ yr^−1^ (Table 1). Compared to the eastern America, Wageningen and western Australia, TP had a lower MNRC (mean: 2.24 g N m^−2^ yr^−1^) (Table 1). The SEM explained 69% (Appendix S2: Fig. S5) of variances in MNRC. Both ANPP_ck_ and N_cr_ had strong positive effects on MNRC. ANPP_ck_ had a positive linear relationship with MNRC (*R*^2^ = 0.78, *F*_1,88_ = 320.11, *P* < 0.001) (Fig. 3c and Appendix S2: Table S4).

### Spatial variations of N-induced C gain

The response of N-induced C gain to N addition was similar to that of N retention capacity (Fig. 4). In Namco steppe, C gain increased to 7.81 ± 5.76 g C m^−2^ yr^−1^ at the N_cr_ of 4 g N m^−2^ yr^−1^, and then gradually leveled off (Fig. 4c). Similarly, C gain of Namco was relatively low in the world (Fig. 5b). The maximum N-induced C gain of global grassland ranged from 4.86 to 583.73 g C m^−2^ yr^−1^, with an average of 56.93 g C m^−2^ yr^−1^ (Table 1). Eastern TP (mean: 53.27 g C m^−2^ yr^−1^) had a higher maximum C gain than the western TP (mean: 12.73 g C m^−2^ yr^−1^), and the IM (mean: 51.49 g C m^−2^ yr^−1^) was similar to that in the eastern TP (Fig. 6c,d; Table 1). Like MNRC, ANPP_ck_ also showed a positive linear relationship with the maximum N-induced C gain (*R*^2^ = 0.82, *F*_1,88_ = 388.59, *P* < 0.001) (Fig. 3d and Appendix S2: Table S4).

### Spatial variations and regulating factors of N_cr_

For global grassland, N_cr_ ranged from 1 to 61.3 g N m^−2^ yr^−1^, with an average of 9.79 g N m^−2^ yr^−1^ (Appendix S2: Table S2). There was a significant difference of N_cr_ on global scale (*F*_7,66_ = 6.38, *P* < 0.001; Table 1). For example, the mean N_cr_ of TP was 5.53 g N m^−2^ yr^−1^, which was significantly lower than that of eastern America, Wageningen and western Australia (Table 1). Environmental factors significantly influenced the variation of N_cr_. In the SEM, all factors could explain 43% of variance in N_cr_ (Appendix S2: Fig. S5). In particular, MAP could positively regulate the N_cr_ directly or indirectly through soil C:N (Appendix S2: Fig. S5). N_cr_ demonstrated positive linear correlations with MAP (*R*^2^ = 0.73, *F*_1,88_ = 239.80, *P* < 0.001; Fig. 3b) and soil C:N (*R*^2^ = 0.65, *F*_1,80_ = 147.71, *P* < 0.001; Appendix S2: Table S4 and Fig. S6c) in the simple regressions.

### Effects of fencing management and grassland types on N_cr_, NRF and MNRC

In our data synthesis, 54 experiments were carried out under the enclosure and the other 35 cases were carried out under grazing (Appendix S2: Table S1). The N_cr_ of enclosure (mean: 8.06 g N m^−2^ yr^−1^) was lower than grazing (mean: 13.21 g N m^−2^ yr^−1^; Appendix S2: Table S5). Fencing management only reduced N_cr_ (*t* = − 2.69, *df* = 87, *P* = 0.01) and had no statistically significant effect on plant NRF (*t* = 1.54, *df* = 86.98, *P* = 0.13) or MNRC (*t* = − 1.04, *df* = 87, *P* = 0.30) (Appendix S2: Table S5).

Grassland types (meadow, steppe and desert steppe) significantly influenced N_cr_ (*F*_2,81_ = 4.72, *P* = 0.01). N_cr_ for meadow, steppe and desert steppe were 6.19, 11.65 and 10.00 g N m^−2^ yr^−1^, respectively (Appendix S2: Table S6). NRF was marginally significant (*F*_2,81_ = 2.73, *P* = 0.07) across the different grassland types, with the highest NRF (mean: 47.19%) in meadow, followed by steppe (mean: 35.20%) and desert steppe (mean: 17.90%; Appendix S2: Table S6).

## Discussion

### N addition altered species composition in alpine grassland

Combining our field experiment with 13 multi-level studies conducted on the Tibetan, we found that functional groups respond similarly to N addition across alpine grasslands: N addition reduced the AGB proportion of forbs (Appendix S2: Fig. S8c), which is similar to Zong, et al.^43^. N addition increased AGB proportion of grasses except sedges (Appendix S2: Fig. S8a,b), consistent with a meta-analysis by Wang, et al.^35^. For legumes, N addition reduced their AGB proportion (Appendix S2: Fig. S8d), which is also demonstrated by Huang, Liu and Zhou^44^ and Xu, et al.^45^.

The above results indicated that the resource allocation strategies differ between species after fertilization^46^. Several reasons may account for these changes. First, ecosystems constrained by N may reach N saturation and plant growth will convert soil N competition to light competition under eutrophication conditions^47^. The dominant species (such as *Asteraceae*) and grasses (*Poaceae*) are competitive in light competition due to their higher stems and dense leaves, thus they could grow faster under conditions with sufficient N and water supply. The small and slow-growing forbs (such as *Boraginaceae*) and sedges (*Cyperaceae*) are weak in light competition because they are at the lower layer in the vertical structure of the community. Therefore, even if short-term N addition could promote their growth, competition on light may lead to inferior species to decrease sharply or disappear eventually^48,49^. Second, legumes can promote plant growth through N fixation with rhizobia under low N level. At high N level, this ability may be inhibited and their inherent advantage over other non-legume species may be diminished^50,51^, but it is beneficial to the growth and reproduction of grasses^52^.

In our field experiment, before and after the N_cr_, biomass of *Asteraceae* changed obviously (Fig. 2a). In addition to excessive N inhibiting the growth of *Asteraceae*, this change may also be related to the stability of plant. The study of Huang, Liu and Zhou^44^ reported that N addition significantly reduced the stability of *Asteraceae*, but had no effect on other species. This suggests that *Asteraceae* should be noted when studying the effects of N addition on plants, as they may be able to indicate the N saturation points in alpine grassland.

Our synthesis revealed that ANPP first increased with N addition and then saturated at N_cr_ (Appendix S2: Fig. S2). This could be illustrated as follows: N addition improves soil N availability through alleviating N limitation and thus boosts plant growth^33,53^, but excess N inputs to the soil may shift limitation factor from N to light^54^, so the ANPP saturated at N_cr_. Moreover, we found the ratio of ANPP_max_ to initial ANPP was approximately 1.67 (the ratio in TP grasslands was about 1.45; Fig. 3a) and this coefficient was greater than the study of LeBauer and Treseder^11^ (1.29). This may be because LeBauer and Treseder^11^ chose ANPP at the highest rate of N addition while we chose ANPP at N_cr_, and the highest rate reduces ANPP due to oversaturation which inhibits plant growth.

### N addition increased N retention and C gain until reached N_cr_

Not all the exogenous N could be retained in the plant pool. NRF would decrease with elevated N and stabilized at N_cr_. A global average of less than half of N addition can be retained by plants (NRF: 39.30%; Table 1). At high altitudes on the Tibetan Plateau, N retention capacity is even lower (NRF: 13.91%; Table 1). The N_cr_ is considered to be the lowest N input that leads a major harmful effect on the sensitive ecological indicator, and it is essential to determine the N_cr_ for alpine grasslands in order to provide an early warning before harmful effects occur in these ecosystems^19,43^ (Fig. 1). When the N addition level is lower than local N_cr_, the ANPP and N retention in the plant will increase with N addition, along with C accumulation. Once the N addition level is higher than the local N_cr_, plant N retention and C gain will reach its maximum value. In previous study, for the alpine steppe, when the N addition level is higher than N_cr_ level (which is about 4 g N m^−2^ yr^−1^), ANPP will reach a maximum, and ecosystems will be saturated, inorganic N will be accumulated^38,55^, the C and N mineralization rate will reach the maximum^12^, the ecosystems function might even turn from a C sink to source^12^. Therefore, it is quite crucial to constrain the reasonable N application level in the grassland restoration management to avoid the soil N pollution and keeping the C sink function for the alpine grassland.

In this study, given the large spatial variability of N_cr_ and MNRC, we found N_cr_ was highly correlated with MAP. This indicates the wetter the environment, the greater N_cr_, which is similar to the previous study of Peng, Chen and Yang^17^. While the initial ANPP is a reliable predictor for MNRC. The significant linear correlation between ANPP_ck_ and MNRC suggests that we might predict the MNRC of local grassland by observing local ANPP (Fig. 3c).

### Fencing management and grassland types influenced N_cr_, NRF and MNRC

Fencing and grazing may affect belowground C-N cycling and thus the uptake of exogenous N by plants^56^. Fencing management is often used to restore degraded grasslands^57^. In our study, we found that the N_cr_ was significantly lower in fencing grassland than in grazing grassland (Appendix S2: Table S5), indicating that grazing grassland might need more N to reach its saturation. In the case of fencing, N was accumulated and recycled in the plant-soil system because no biomass was harvested and relatively little exogenous N was required^56^. For the grazing sample plots, the plants consumed by livestock were comparable to the biomass harvested, so N did not accumulate in the soil and more N was required for the plants to reach saturation of ANPP^35,57^.

Although Peng, Chen and Yang^17^ also compared the N_cr_ of biomass with harvest and without harvest, there was no significant differences in N_cr_ between the above two. The distinct results between our work and Peng, Chen and Yang^17^ can be explained in two aspects: First, for some experimental cases of fencing in the growing season and grazing in non-growing season, we treated them as the fencing sample plots, while Peng, Chen and Yang^17^ considered them as the grazing sample plots. We applied our own criteria to the case of Peng, Chen and Yang^17^ and found a marginal significance (*t* = − 1.91, *df* = 53, *P* = 0.06) between fencing and grazing. Consequently, the inconsistency of criteria for judging the experimental cases may have an account for the inconsistent results. Second, we collected 89 cases and Peng, Chen and Yang^17^ collected 55 cases, the larger amount of data may have caused the difference between fencing and grazing.

Grassland type also potentially influenced N_cr_. Previous works have shown that the N_cr_ of a semiarid grassland was 9.17 g N m^−2^ yr^−1^^58^ and a temperate grassland was 10.5 g N m^−2^ yr^−1^^24^. Our data synthesis also revealed that the steppe had a higher N_cr_ (11.65 g N m^−2^ yr^−1^) and MNRC (4.10 g N m^−2^ yr^−1^) than meadow (N_cr_: 6.19 g N m^−2^ yr^−1^, MNRC: 2.92 g N m^−2^ yr^−1^; Appendix S2: Table S6). This suggests that steppe might require more N to reach its N saturation when compared to meadow and desert steppe, steppe had the highest MNRC, which was mainly attributed to its highest ANPP (Appendix S2: Table S6). From another perspective, most of the meadow sites were located on the Tibetan Plateau, where the relatively low precipitation further reduced the N_cr_ (Fig. 3b).

### Implications

Under N limitation, N addition promoted the function of C and N absorption in grassland^11,15^. N application exceeding grassland carrying capacity will reduce grassland biodiversity and may reverse grassland C sink function^12,16^, alter species composition, groundwater resources and threaten ecological security^20^. Therefore, a better understanding of N retention capacity in plant pool and reasonable assessment of N_cr_ is important for ecosystem.

Based on a six-year multi-level (0, 1, 2, 4, 8 and 16 g N m^−2^ yr^−1^) N fertilization experiment and a data synthesis included 89 cases of N addition experiments, plant N retention showed a universal nonlinear pattern with N addition. Although N addition increased ANPP and N retention, the increasing trend disappeared at N_cr_. The fixed ratio of ANPP_max_ to ANPP_ck_ (about 1.67) indicated that the N retention of grassland does not increase linearly with N input (Fig. 3a). When external N addition exceeded N_cr_, the exogenous N stored by plants reached its maximum. The positive MNRC-ANPP_ck_ linear relationship revealed that the MNRC might be directly predicted by initial ANPP (Fig. 3c). Compared with other grassland in the world, Tibetan alpine grassland had the lowest MNRC and N_cr_, implying the lowest N deficiency (Fig. 5; Table1).

Given the current level of atmospheric N deposition on the TP (0.32 to 1.23 g N m^−2^ yr^−1^)^31,32^, the N retention and C gain of Namco steppe ranged from 0.07 to 0.26 g N m^−2^ yr^−1^ (Fig. 4a) and 1.00 to 3.66 g C m^−2^ yr^−1^ (Fig. 4c), respectively. For the entire TP grassland covering about 1.54 million km^2^, TP alpine grasslands could retain 0.33–1.21 Tg N yr^−1^ (0.22–0.79 g N m^−2^ yr^−1^; Fig. 4b) and gain 5.65–20.11 Tg C yr^−1^ (3.67–13.06 g C m^−2^ yr^−1^; Fig. 4d). With the aggravation of N deposition, the capacity of N and C absorption over the TP grasslands will increase with the increase of ANPP until it reaches the N_cr_. Considering the average N_cr_ of the whole TP was 5.53 g N m^−2^ yr^−1^, N retention and C gain of the TP grassland will increase to 4.41 Tg N yr^−1^ (2.86 g N m^−2^ yr^−1^; Fig. 4b) and 64.39 Tg C yr^−1^ (41.81 g C m^−2^ yr^−1^; Fig. 4d) in the future.

The key parameters obtained from this study include the ratio of ANPP_max_ to ANPP_ck_, N_cr_, NRF, MNRC, maximum N-induced C gain and their climate driven factors. These parameters are crucial to constrain the C-N coupling model for further constructing robust predictions of N input on ecosystem C balance and find out the optimal N application rate for N resource management in the restoration of degraded grasslands on the TP.

## Supplementary Information

Supplementary Information.

## Data Availability

All data generated or analysed during this study are included in this published article (and its supplementary information files).

## Abbreviations

AGB: Aboveground biomass
ANPP: Aboveground net primary productivity
BNPP: Belowground net primary productivity
IM: Inner Mongolia
MAP: Mean annual precipitation
MAT: Mean annual temperature
MNRC: Maximum N retention capacity
N_cr_: Critical N rate
NRF: N retention fraction
SEM: Structural equation modeling
TP: Tibetan Plateau
