# Supplementary material for: Plant nitrogen retention in alpine grasslands of the Tibetan Plateau under multi-level nitrogen addition
Source: Sci Rep. 2023 Jan 17;13:877. doi: 10.1038/s41598-023-27392-y (PMC9845361; doi:10.1038/s41598-023-27392-y)
Supplement: Supplementary file 1 — Supplementary Information. [file 41598_2023_27392_MOESM1_ESM.pdf]

Plant nitrogen retention in alpine grasslands of the Tibetan Plateau under multi-level nitrogen addition

Jiaoneng Yu[1,2,5], Xu-Ri[2,5,*], Songbo Qu[1,2], Fengzi Li[2,3], Da Wei[2,4], Almaz Borjigidai[1,*]

[1]Key Laboratory of Ethnomedicine, Ministry of Education, Minzu University of China, Beijing 100081, China

[2]State Key Laboratory of Tibetan Plateau Earth System, Resources and Environment, Institute of Tibetan Plateau Research, Chinese Academy of Sciences, Beijing 100101, China

[3]Inner Mongolia Academy of Forestry Sciences, Inner Mongolia, Hohhot 010010, China

[4]Key Laboratory of Mountain Surface Processes and Ecological Regulation, Institute of Mountain Hazards and Environment, Chinese Academy of Sciences, Chengdu 610041, China

[5]These authors contributed equally to this work

[*]Corresponding authors:

Xu-Ri. Email: xu-ri@itpcas.ac.cn;

Almaz Borjigidai. Email: almaz_b@muc.edu.cn

Appendix S1

The appendix contains supplementary figures and tables related to our field experiment in Namco alpine steppe.

Supplementary Tables

Table S1. List of species within the quadrats (50 × 50 cm) across 2011 to 2013, 2105 and 2017 in Namco steppe.

Functional groups	Species or genus	LHT	Proportion (%)
Asteraceae (Forbs)	*Ajania przewalskii*	P	28.89
	Artemisia stracheyi	P	
	Artemisia wellbyi	P	
	Aster semiprostratus	P	
	Leontopodium pusillum	P	
	Saussurea japonica	P	
Poaceae (Grasses)	*Festuca ovina*	P	7.40
	Poa litwinowiana	P	
	Puccinellia distans	P	
	Stipa purpurea	P	
	Stipa tianschanica	P	
	Trisetum tibeticum	P	
Leguminosae (Legumes)	*Astragalus arnoldii*	P	5.20
	Oxytropis biflora	P	
	Oxytropis falcata	P	
	Oxytropis glacialis	P	
	Oxytropis microphylla	P	
	Oxytropis stracheyana	P	
Rosaceae (Forbs)	*Potentilla anserina*	P	9.26
	Potentilla bifurca	P	
	Potentilla multiceps	P	
	Potentilla saundersiana	P	
	Sibbaldia adpressa	P	
Boraginaceae (Forbs)	*Eritrichium*	AB	1.76
	Microula tibetica	B	
Caryophyllaceae (Forbs)	*Arenaria bryophylla*	P	5.30
	Stellaria arenaria	P	
Cyperaceae (Sedges)	*Carex moorcroftii*	P	8.20
	Kobresia macrantha	P	
Labiatae (Forbs)	*Dracocephalum heterophyllum*	P	10.54
Primulaceae (Forbs)	*Androsace tapete*	P	19.63
Scrophulariaceae (Forbs)	*Pedicularis alaschanica*	P	2.61
Others	*Ephedra monosperma*	P	1.22
	Pleurospermum camtschaticum	P	

Functional groups	Species or genus	LHT	Proportion (%)
	Gentiana suborbisepala	A	
	Dimorphostemon glandulosus	A	
	Gentiana veitchiorum	P	
	Lepidium capitatum	AB	
	Chenopodium album	A	
	Salsola collina	A	
	Incarvillea younghusbandii	P	
	Unknown		

Note: Proportion (%) represents total aboveground biomass contribution of each functional group. Life history trait (LHT) are indicated by P, perennial; A, annual; AB, annual & biennial. The "unknown" represents a small number of unidentified species.

Table S2. Two-way ANOVA results for the effects of nitrogen (N) fertilization, year and their interactions on species aboveground biomass (AGB) and proportion of *Asteraceae* (AS), *Primulaceae* (PR), *Labiatae* (LA), *Rosaceae* (RO), *Poaceae* (PO), *Leguminosae* (LE), *Cyperaceae* (CY), *Caryophyllaceae* (CA), *Boraginaceae* (BO) and *Scrophulariaceae* (SC). The six levels of N fertilization rate were 0, 1, 2, 4, 8 and 16 g N m^{-2} yr^{-1}. Biomass was collected from 2011 to 2013, 2015 and 2017 in Namco steppe. Bold values indicate significance (P <0.05).

		AGB (g m^{-2})			AGB proportion (%)		
Family	Term	*df*	*F*	*P*	*df*	*F*	*P*
AS	N fertilization	5, 60	3.161	**0.013**	5, 60	2.669	**0.03**
	Year	4, 60	2.814	**0.033**	4, 60	5.299	**0.001**
	N × Year	20, 60	1.31	0.209	20, 60	0.748	0.761
PR	N fertilization	5, 60	0.489	0.783	5, 60	0.356	0.877
	Year	4, 60	3.646	**0.01**	4, 60	4.27	**0.004**
	N × Year	20, 60	0.497	0.958	20, 60	0.413	0.984
LA	N fertilization	5, 60	2.43	**0.045**	5, 60	2.739	**0.027**
	Year	4, 60	26.444	**<0.001**	4, 60	12.524	**<0.001**
	N × Year	20, 60	2.125	**0.013**	20, 60	2.549	**0.003**
RO	N fertilization	5, 60	2.637	**0.032**	5, 60	0.936	0.464
	Year	4, 60	4.196	**0.005**	4, 60	0.713	0.586
	N × Year	20, 60	2.744	**0.001**	20, 60	1.366	0.176
PO	N fertilization	5, 60	1.371	0.248	5, 60	1.739	0.139
	Year	4, 60	3.505	**0.012**	4, 60	2.621	**0.044**
	N × Year	20, 60	0.629	0.875	20, 60	0.382	0.99
LE	N fertilization	5, 60	1.061	0.391	5, 60	1.126	0.356
	Year	4, 60	1.712	0.159	4, 60	2.001	0.106
	N × Year	20, 60	1.114	0.361	20, 60	0.772	0.735
CY	N fertilization	5, 60	2.815	**0.024**	5, 60	3.574	**0.007**
	Year	4, 60	20.051	**<0.001**	4, 60	15.418	**<0.001**
	N × Year	20, 60	2.276	**0.008**	20, 60	2.702	**0.002**
CA	N fertilization	5, 60	0.544	0.742	5, 60	0.44	0.819
	Year	4, 60	2.314	0.068	4, 60	2.557	**0.048**
	N × Year	20, 60	0.888	0.603	20, 60	0.888	0.602
BO	N fertilization	5, 60	1.248	0.298	5, 60	0.871	0.506
	Year	4, 60	2.865	**0.031**	4, 60	3.103	**0.022**
	N × Year	20, 60	1.704	0.058	20, 60	1.281	0.227
SC	N fertilization	5, 60	1.109	0.365	5, 60	0.846	0.523
	Year	4, 60	2.433	0.057	4, 60	2.711	**0.038**

		AGB (g m^{-2})			AGB proportion (%)		
Family	Term	*df*	*F*	*P*	*df*	*F*	*P*
	N × Year	20, 60	0.561	0.924	20, 60	0.577	0.914
Total	N fertilization	5, 60	1.505	0.202	-	-	-
	Year	4, 60	7.065	**<0.001**	-	-	-
	N × Year	20, 60	1.134	0.342	-	-	-

Table S3 One-way ANOVA results for the effects of nitrogen (N) fertilization on foliar carbon (C) content, N content and C:N ratio of *Asteraceae* (AS), *Primulaceae* (PR), *Labiatae* (LA), *Rosaceae* (RO), *Poaceae* (PO), *Leguminosae* (LE), *Cyperaceae* (CY), *Caryophyllaceae* (CA), *Boraginaceae* (BO) and *Scrophulariaceae* (SC) in 2011. Bold values indicate significance (P <0.05).

	C content (%)			N content (%)			C:N ratio		
Family	*df*	*F*	*P*	*df*	*F*	*P*	*df*	*F*	*P*
AS	5, 12	1.991	0.152	5, 12	2.996	0.055	5, 12	1.974	0.155
PR	5, 7	2.09	0.182	5, 7	7.58	**0.01**	5, 7	8.414	**0.007**
LA	5, 11	3.654	**0.034**	5, 11	8.096	**0.002**	5, 11	7.611	**0.003**
RO	5, 11	1.102	0.413	5, 11	1.906	0.173	5, 11	2.201	0.128
PO	5, 12	1.414	0.288	5, 12	1.877	0.172	5, 12	1.509	0.258
LE	5, 7	1.309	0.359	5, 7	0.56	0.729	5, 7	0.592	0.709
CY	5, 12	0.619	0.688	5, 12	4.606	**0.014**	5, 12	3.841	**0.026**
CA	5, 7	2.463	0.136	5, 7	3.015	0.091	5, 7	2.49	0.133
BO	4, 5	1.3	0.383	4, 5	5.426	**0.046**	4, 5	3.712	0.091
SC	2, 4	0.589	0.597	2, 4	2.553	0.193	2, 4	1.243	0.38
Total	5, 138	0.801	0.551	5, 138	9.075	**<0.001**	5, 138	7.782	**<0.001**

Table S4 One-way ANOVA results for the effects of nitrogen (N) fertilization on aboveground net primary productivity (ANPP), belowground net primary productivity (BNPP) and their root:shoot ratio in 2011, 2013, 2015 and 2017.

	ANPP (g m^{-2} yr^{-1})			BNPP (g m^{-2} yr^{-1})			Root:shoot ratio		
Year	*df*	*F*	*P*	*df*	*F*	*P*	*df*	*F*	*P*
2011	5, 12	1.42	0.286	5, 12	0.535	0.746	5, 12	1.24	0.35
2013	5, 12	0.584	0.713	5, 12	0.476	0.788	5, 12	1.217	0.359
2015	5, 12	1.986	0.153	5, 12	1.733	0.202	5, 12	1.504	0.26
2017	5, 12	0.73	0.614	5, 12	0.906	0.509	5, 12	1.013	0.452

Supplementary Figures

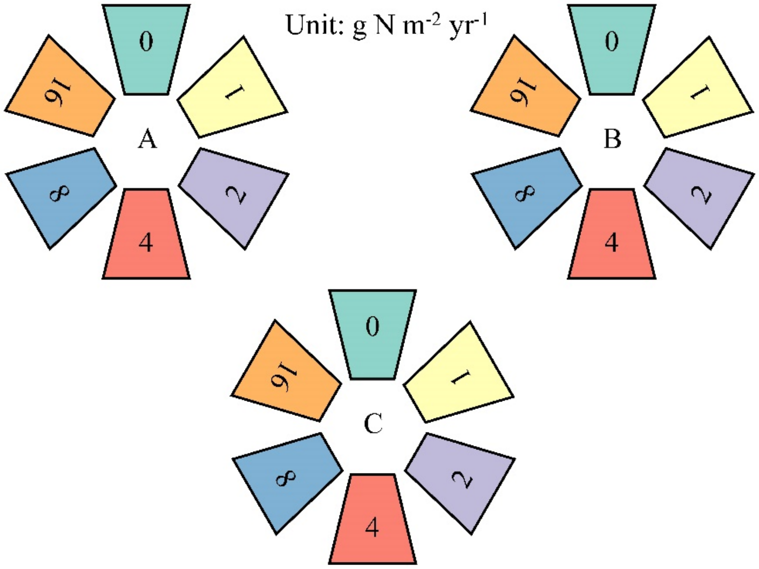

Figure S1. Maps of the field experiment site. Three homogenous plots were randomly arranged as replicates at Namco steppe in May 2010, and six subplots (~13 m^2, with a 2 m buffer zone between each adjacent subplot) were fertilized with six levels of 0, 1, 2, 4, 8 and 16 g N m^{-2} yr^{-1}, respectively.

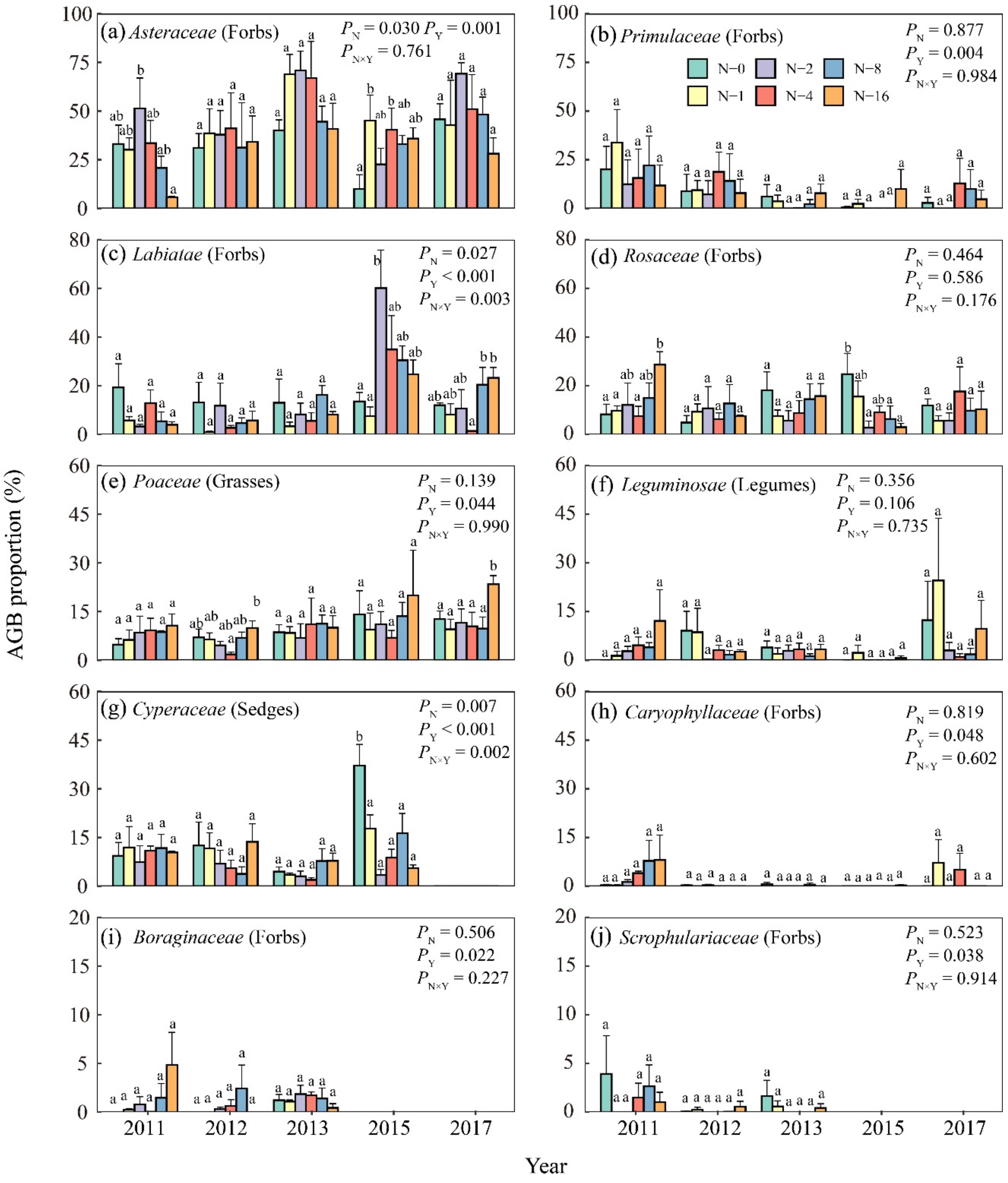

Figure S2. Aboveground biomass (AGB) proportion of species from ten families in response to long term N fertilization rate. Data analysis was based on the field experiment (Namco steppe). Biomass was collected from 2011 to 2013, 2015, and 2017 in Namco. Data are shown as mean ± SE (*n* = 3). Bars with different letters indicate

significant differences ($P < 0.05$) by Duncan's new multiple range test among fertilization levels. Full statistical results for two-way ANOVA (N, N fertilization; Y, year) are presented in Appendix S1: Table S2.

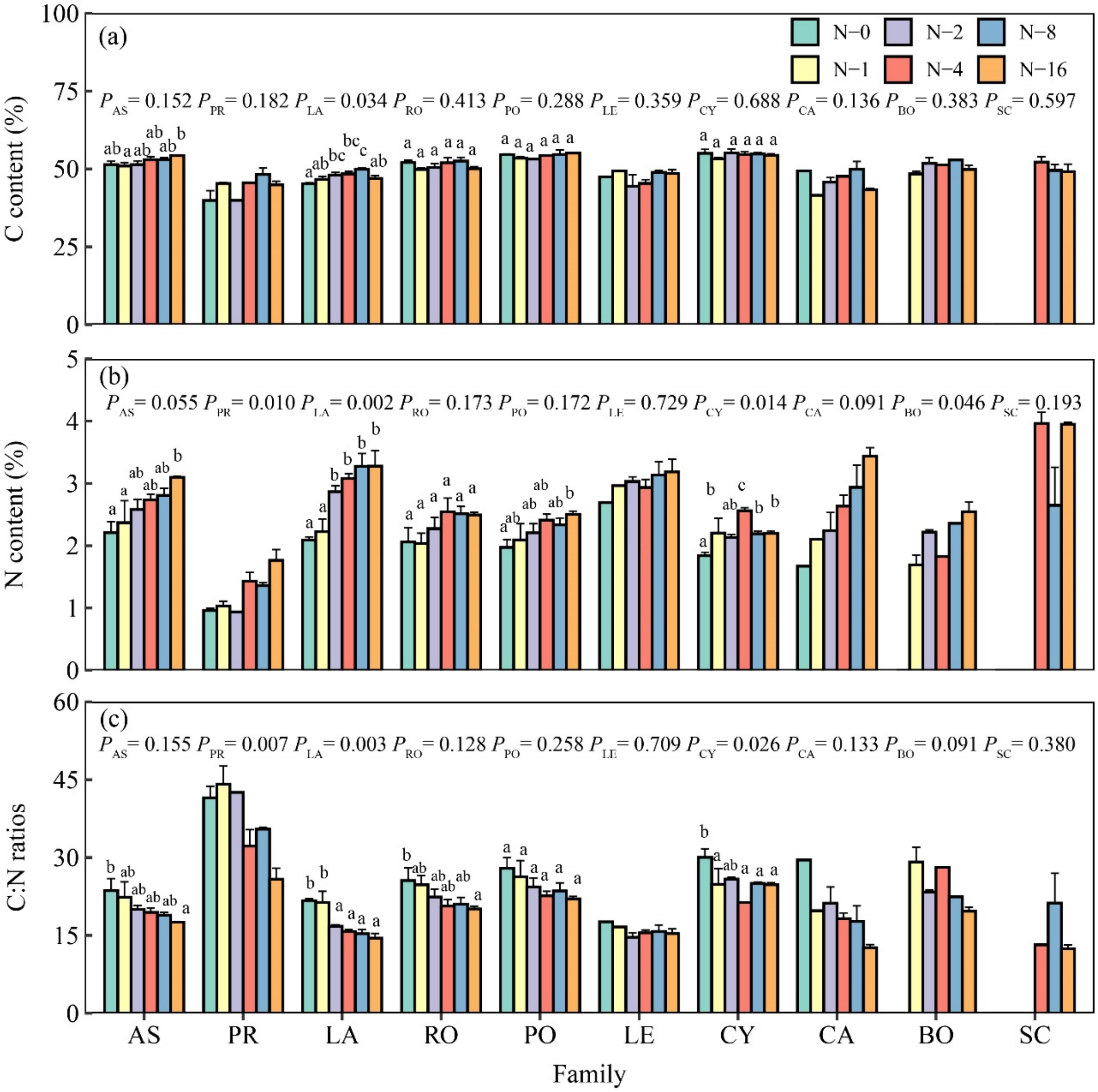

Figure S3. Foliar carbon (C), N content and C:N ratios of species from ten families in response to N fertilization rate in Namco steppe. AS, *Asteraceae*; PR, *Primulaceae*; LA, *Labiatae*; RO, *Rosaceae*; PO, *Poaceae*; LE, *Leguminosae*; CY, *Cyperaceae*; CA, *Caryophyllaceae*; BO, *Boraginaceae*; SC, *Scrophulariaceae*. Data analysis was based on the field experiment (Namco steppe). Data are shown as mean ± SE. Bars with different letters indicate significant differences ($P < 0.05$) by Duncan's new multiple range test among fertilization levels. Full statistical results for one-way ANOVA are presented in Appendix S1: Table S3.

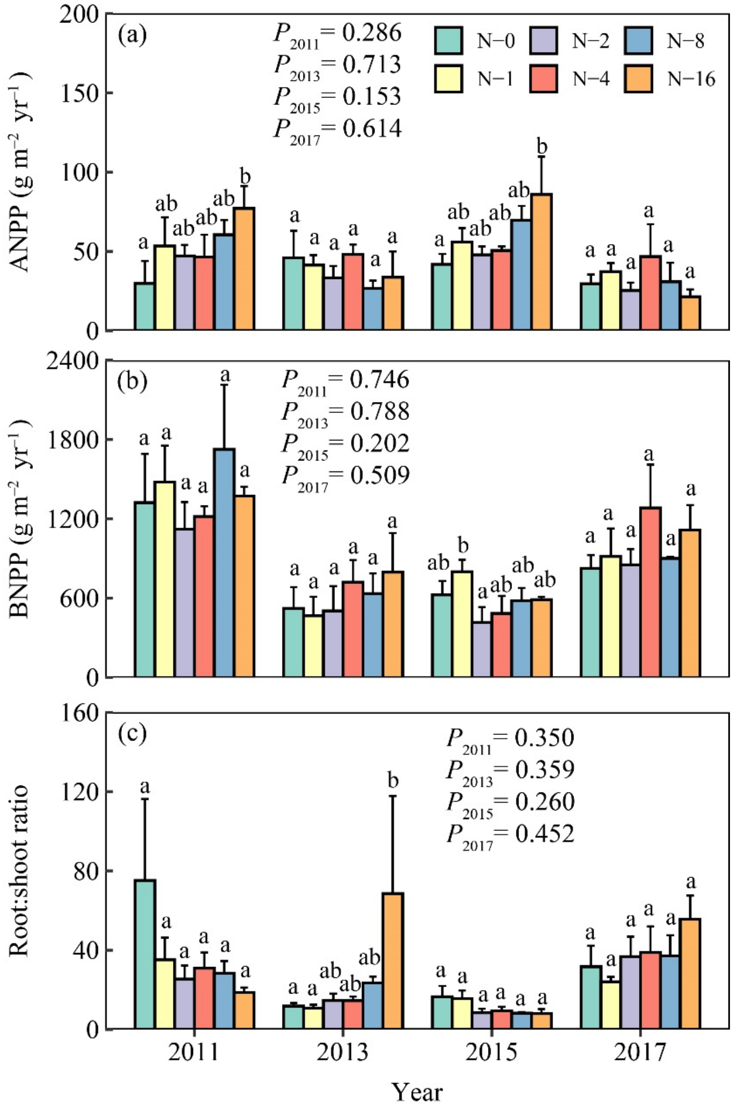

Figure S4. The effect of N fertilization on species above- and belowground net primary productivity as well as root:shoot ratio in Namco steppe. Panels represent the (a) aboveground net primary productivity (ANPP), (b) belowground net primary productivity (BNPP) and their (c) root:shoot ratio during 2011, 2013, 2015 and 2017. Data are shown as mean ± SE (n = 3). Bars with different letters indicate significant differences (P < 0.05) by Duncan's new multiple range test among fertilization levels. Full statistical results for one-way ANOVA are presented in Appendix S1: Table S4.

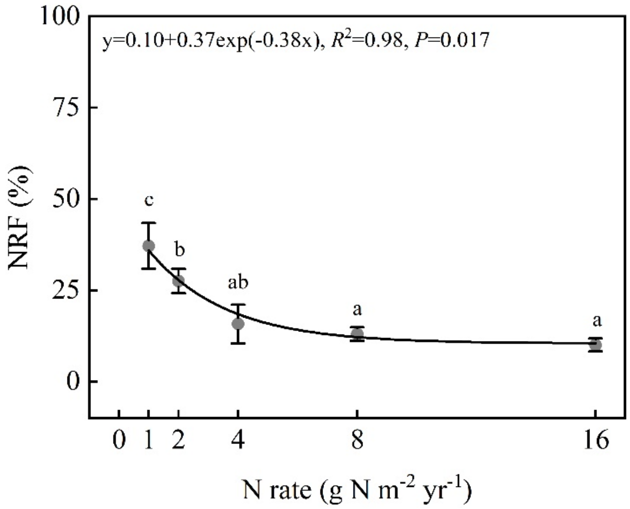

Figure S5. Effects of N fertilization on N retention fraction (NRF) of Namco field experiment in 2011 and 2015. Simple regression analysis was implemented. Regression equation, coefficient of determination (R^2) and P-value are shown. Bars with different letters indicate significant differences ($P < 0.05$) by Duncan's new multiple range test among fertilization levels.

Appendix S2

The appendix includes supplementary tables and figures related to the data synthesis section.

Supplementary Tables

Table S1. Detailed information for the 89 experimental cases and the reference sources are shown in the Source 1.

Case	LAT	LONG	ASL	MAT	MAP	Soil C:N	Fencing management	Grassland type	Region	Source
case 1	31.38	90.23	4590	-0.811	429	10.93	Fencing	Meadow	Bangoin, CN	Zong et al., 2019
case 2	30.85	91.08	4333	-3.21	419	16.17	Grazing	Meadow	Damxung, CN	Zong et al., 2016
case 3	30.85	91.08	4333	-3.21	419	16.17	Fencing	Meadow	Damxung, CN	Zong et al., 2014
case 4	30.8	91.1	4333	-3.21	419	12.46	Grazing	Meadow	Damxung, CN	Zong et al., 2012
case 5	32.37	82.27	4520	-5.59	83	10.66	Fencing	Desert steppe	Gerze, CN	Zong et al., 2019
case 6	31.57	92.57	4570	-4.145	520	18.55	Fencing	Meadow	Nagqu, CN	Zong et al., 2019
case 7	31.44	92.02	4500	-0.74	438	12.56	Fencing	Meadow	Nagqu, CN	Yan et al., 2018
case 8	30.78	90.97	4730	-0.98	413	10.64	Grazing	Steppe	Namco, CN	These study
case 9	30.78	90.97	4730	-0.98	413	10.64	Grazing	Steppe	Namco, CN	Liu et al., 2013
case 10	31.78	87.23	4580	1.27	233	10.87	Fencing	Steppe	Nyima, CN	Zong et al., 2019
case 11	37.6	101.2	3240	-1.7	469	-	Fencing	Meadow	Haibei, CN	Song et al., 2012b
case 12	37.3	100.25	3290	0.06	383	8.20	Fencing	Steppe	Haibei, CN	Peng et al., 2017
case 13	37.6	101.3	-	-0.71	469	5.35	Fencing	Meadow	Haibei, CN	Fang et al., 2014
case 14	37.6	101.3	-	-0.71	469	5.35	Fencing	Meadow	Haibei, CN	Fang et al., 2014
case 15	37.6	101.3	-	-0.71	469	5.35	Fencing	Meadow	Haibei, CN	Fang et al., 2014
case 16	37.6	101.3	3220	-0.712	469	5.68	Fencing	Meadow	Haibei, CN	Zhu et al., 2011
case 17	37.6	101.3	3220	-0.712	469	5.68	Fencing	Meadow	Haibei, CN	Zhu et al., 2011
case 18	37.6	101.3	3220	-0.71	469	5.68	Fencing	Meadow	Haibei, CN	Zhu et al., 2011
case 19	34.92	102.88	2950	2.05	583	7.56	Fencing	Meadow	Lanzhou, CN	Sun et al., 2015
case 20	36	101.9	3500	4.62	483	5.67	Fencing	Meadow	Walaka experimental site, CN	Liu et al., 2012
case 21	35.58	101.53	3500	-4.28	596	10.26	Grazing	Meadow	Maqu, CN	Zhang, 2014
case 22	35.97	101.88	3500	4.62	483	10.26	Fencing	Meadow	Maqu, CN	Li et al., 2017
case 23	35.97	101.88	3500	1.2	483	-	Fencing	Meadow	Maqu, CN	Xu et al., 2015a
case 24	36	101.9	3500	4.62	483	5.67	Fencing	Steppe	Maqu, CN	Qiu and Luo, 2004
case 25	35.97	101.88	3500	4.62	483	10.26	Fencing	Meadow	Maqu, CN	Zhou et al., 2017
case 26	32.98	103.67	-	1.75	756	11.76	Fencing	Meadow	Pansong County, CN	Chen and Chen, 2017
case 27	32.8	102.6	3500	2.79	713	10.57	Grazing	Meadow	Hongyuan County, CN	Song et al., 2017
case 28	42.03	116.28	1324	2.52	428	7.24	Fencing	Steppe	Duolun County, CN	Song et al., 2012a
case 29	42.03	116.28	1324	2.52	428	7.23	Grazing	Steppe	Duolun County, CN	Liu et al., 2014

Case	LAT	LONG	ASL	MAT	MAP	Soil C:N	Fencing management	Grassland type	Region	Source
case 30	42.03	116.28	1324	2.52	428	7.24	Grazing	Steppe	Duolun County, CN	Hao et al., 2017
case 31	42.03	116.28	1324	2.52	428	7.24	Fencing	Steppe	Duolun County, CN	Zheng et al., 2019
case 32	42	116.3	1324	2.52	428	7.24	Fencing	Steppe	Duolun County, CN	Song et al., 2011
case 33	42	116.3	1324	2.52	428	7.24	Grazing	Steppe	Duolun County, CN	Tian et al., 2016
case 34	42	116.3	1324	2.52	428	7.24	Fencing	Steppe	Duolun County, CN	Fang et al., 2012
case 35	42.11	115.49	1450	3.165	381	12.17	Fencing	Steppe	Taibus County, CN	Sheng et al., 2018
case 36	43.6	116.7	1250	1.62	328	8.82	Fencing	Steppe	Xilin River Basin, CN	Bai et al., 2010
case 37	43.6	116.7	1250	1.62	328	8.82	Fencing	Steppe	Xilin River Basin, CN	Bai et al., 2010
case 38	43.2	116.2	1255	1.4	325	11.74	Fencing	Steppe	Xilin River Basin, CN	Zhang et al., 2015
case 39	43.2	116.2	1255	1.4	325	11.95	Fencing	Steppe	Xilin River Basin, CN	Zhang et al., 2015
case 40	43.38	116.7	1200	1.62	328	11.84	Fencing	Steppe	Xilingol League, CN	Wang et al., 2019
case 41	44.17	116.47	-	1.82	289	10.48	Grazing	Steppe	Xilingol League, CN	He et al., 2015
case 42	41.6	114.9	-	3.93	372	8.44	Fencing	Steppe	Xilingol League, CN	He et al., 2009
case 43	48.93	119.69	-	-1.24	342	12.45	Fencing	Meadow	Ewenki Autonomous Banner, CN	Li, 2020
case 44	49.35	120.12	-	-2.05	374	-	Grazing	Steppe	Hailar District, CN	He et al., 2015
case 45	39.78	108.66	-	6.62	306	7.89	Fencing	Desert steppe	Ordos City, CN	Li, 2020
case 46	41.5	118.5	-	5.118	481	-	Fencing	Abandoned farmland	Ulan Buton steppe, CN	Xu et al., 2015b
case 47	41.5	118.5	-	5.118	481	-	Fencing	Steppe	Ulan Buton steppe, CN	Xu et al., 2015b
case 48	41.5	118.5	-	5.118	481	-	Fencing	Steppe	Ulan Buton steppe, CN	Xu et al., 2015b
case 49	41.5	118.5	-	5.118	481	-	Fencing	Steppe	Ulan Buton steppe, CN	Xu et al., 2015b
case 50	41.5	118.5	-	5.118	481	-	Fencing	Steppe	Ulan Buton steppe, CN	Xu et al., 2015b
case 51	41.8	111.9	1450	3.54	225	8.44	Fencing	Desert steppe	Ulanqab, CN	Gao et al., 2018
case 52	41.3	111.22	-	3.15	284	8.43	Grazing	Desert steppe	Xilamuren District, CN	He et al., 2015
case 53	44.4	122.9	-	6.58	436	10.10	Fencing	Steppe	Jilin, CN	Sun et al., 2014
case 54	36.2	106.4	1800	7.08	432	7.97	Grazing	Steppe	Guyuan County, CN	Zhao et al., 2017
case 55	38.8	110.4	1081	7.79	417	10.92	Fencing	Steppe	Shenmu County, CN	Han et al., 2011
case 56	38.8	110.4	1081	7.79	417	9.97	Fencing	Steppe	Shenmu County, CN	Han et al., 2011
case 57	43	122.4	260	6.94	494	6.69	Fencing	Steppe	Shenyang, CN	Li et al., 2009

Case	LAT	LONG	ASL	MAT	MAP	Soil C:N	Fencing management	Grassland type	Region	Source
case 58	41.7	115.7	1475	3.22	396	8.08	Fencing	Steppe	Guyuan County, CN	Chen et al., 2016
case 59	42.89	83.71	2500	-2.913	267	10.51	Fencing	Steppe	Xinjiang, CN	Li et al. 2015
case 60	26	103	1350	10.22	872	8.25	Fencing	Steppe	Kunming, CN	Zhang et al., 2004
case 61	42	-93.8	-	8.9	853	12.17	Grazing	Steppe	Boone County, Iowa, USA	Heggenstaller et al., 2009
case 62	42	-93.8	-	8.9	853	12.17	Grazing	Steppe	Boone County, Iowa, USA	Heggenstaller et al., 2009
case 63	42	-93.8	-	8.9	853	12.17	Grazing	Steppe	Boone County, Iowa, USA	Heggenstaller et al., 2009
case 64	42	-93.8	-	8.9	853	12.17	Grazing	Steppe	Boone County, Iowa, USA	Heggenstaller et al., 2009
case 65	40.4	-105.7	3500	0.22	656	13.88	Grazing	Meadow	Colorado, USA	Bowman et al., 2006
case 66	46.8	-100.9	-	5.91	426	6.35	Grazing	Steppe	Mandan, USA	Lorenz and Rogler, 1972
case 67	42.4	-85.6	284	8.97	936	8.16	Grazing	Steppe	Michigan, USA	Ruan et al., 2016
case 68	45.4	-93.2	-	6.28	744	13.22	Fencing	Abandoned farmland	Minnesota, USA	Tilman, 1987; Tilman, 1993
case 69	45.4	-93.2	-	6.28	744	13.22	Fencing	Abandoned farmland	Minnesota, USA	Tilman, 1987; Tilman, 1993
case 70	45.4	-93.2	-	6.28	744	13.22	Fencing	Abandoned farmland	Minnesota, USA	Tilman, 1987; Tilman, 1993
case 71	45.4	-93.2	-	6.28	744	13.22	Fencing	Abandoned farmland	Minnesota, USA	Tilman, 1987; Tilman, 1993
case 72	41.8	-72.3	-	8.95	1249	15.66	Grazing	Steppe	Storrs, USA	Collins and Allinson, 2004
case 73	41.8	-72.3	-	8.95	1249	15.95	Grazing	Steppe	Storrs, USA	Collins and Allinson, 2004
case 74	-37.85	147.58	-	14.76	678	14.08	Fencing	Steppe	Athlone, AU	Gourley et al., 2017
case 75	-34.6	116.2	-	1.4	325	15.65	Grazing	Steppe	AU	Bolland and Guthridge, 2007
case 76	-34.2	115.2	-	16.91	1044	13.04	Grazing	Steppe	Margaret River, AU	Bolland and Guthridge, 2007
case 77	-37.85	147.58	-	14.76	678	12.62	Fencing	Steppe	Gippsland, AU	Gourley et al., 2017
case 78	-36.4	145.2	-	15.02	525	11.92	Fencing	Steppe	Tatura, AU	Gourley et al., 2017
case 79	-33.9	18.8	177	16.94	675	6.21	Grazing	Steppe	Elsenburg	Labuschagne et al., 2013

Case	LAT	LONG	ASL	MAT	MAP	Soil C:N	Fencing management	Grassland type	Region	Source
case 80	58.6	23.6	-	6.51	596	9.09	Grazing	Meadow	Laelatu, EE	Niinemets and Kull, 2005
case 81	36.9	139.9	-	11.46	1396	14.19	Grazing	Meadow	Nasu, JP	Mori and Hojito, 2011
case 82	-40.3	175.8	200	11.84	1142	12.40	Grazing	Steppe	NZ	Hoogendoorn et al., 2017
case 83	50.4	6.9	480	8.09	855	13.94	Grazing	Steppe	Rengen, DE	Anger et al., 2003
case 84	-38.2	142.9	-	13.33	796	12.87	Grazing	Steppe	Victoria, GB	McKenzie et al., 1999
case 85	-38.5	143.1	-	13.51	993	12.87	Grazing	Steppe	Victoria, GB	McKenzie et al., 1999
case 86	52	5.7	-	9.63	799	18.55	Grazing	Steppe	Wageningen, NL	Schröder et al., 2010
case 87	52	5.7	-	9.63	799	18.13	Grazing	Steppe	Wageningen, NL	Schröder et al., 2010
case 88	52	5.7	-	9.63	799	13.81	Grazing	Steppe	Wageningen, NL	Schils et al., 2008
case 89	52	5.7	-	9.63	799	13.81	Grazing	Steppe	Wageningen, NL	Schils et al., 2008

Note: Abbreviations: LAT, latitude; LONG, longitude; ASL, altitude; MAT, mean annual temperature; MAP, mean annual precipitation.

Country codes: CN, China; USA, America; AU, Australia; GB, Britain; NL, Netherlands; EE, Estonia; JP, Japan; DE, Germany.

Table S2. The key parameters obtained from the 89 experimental cases.

Case	N_{cr} (g N m^{-2} yr^{-1})	$ANPP_{ck}$ (g m^{-2} yr^{-1})	$ANPP_{max}$ (g m^{-2} yr^{-1})	N $content_{ck}$ (%)	N content (%)	C $content_{ck}$ (%)	C content (%)	NRF (%)	MNRC (g N m^{-2} yr^{-1})	Maximum N-induced C gain (g C m^{-2} yr^{-1})
case 1	10	44.40	79.35	1.66	2.12	44.04	44.47	9.44	0.94	15.73
case 2	4	47.22	98.06	1.66	2.01	44.04	45.90	29.77	1.19	24.21
case 3	4	68.85	113.73	1.66	2.01	44.04	45.90	28.68	1.15	21.88
case 4	4	48.00	58.00	1.66	2.12	44.04	44.47	9.27	0.37	5.48
case 5	20	23.14	38.94	1.66	2.62	44.04	43.23	3.18	0.64	6.65
case 6	5	119.35	189.97	1.66	2.01	44.04	45.90	36.88	1.84	34.64
case 7	4	48.85	109.85	1.66	2.12	44.04	44.47	35.03	1.40	28.91
case 8	4	35.79	48.51	1.56	2.31	45.73	42.77	15.75	0.63	7.81
case 9	4	29.76	46.48	1.56	2.31	45.73	42.77	15.92	0.64	9.67
case 10	10	27.38	38.04	1.66	2.12	44.04	44.47	3.51	0.35	4.86
case 11	7.5	274.00	337.16	1.66	2.01	44.04	45.90	34.58	2.59	29.26
case 12	8	136.45	260.85	1.36	1.81	44.04	44.47	35.82	2.87	55.90
case 13	4	356.00	391.00	1.66	2.01	44.04	45.90	49.05	1.96	22.70
case 14	1	356.00	397.00	1.66	2.01	44.04	45.90	208.27	2.08	25.45
case 15	2	356.00	405.00	1.66	2.01	44.04	45.90	112.19	2.24	29.12
case 16	1	349.80	431.30	1.66	2.01	44.04	45.90	287.67	2.88	43.92
case 17	1	349.80	444.30	1.66	2.01	44.04	45.90	313.86	3.14	49.89
case 18	2	349.80	447.10	1.66	2.01	44.04	45.90	159.75	3.20	51.18
case 19	15	330.43	516.26	1.66	2.31	44.04	43.18	42.96	6.44	77.42
case 20	19.1	500.00	900.00	1.66	2.62	44.04	43.23	80.06	15.29	168.93
case 21	10	354.80	462.40	2.07	2.31	44.04	43.18	37.09	3.71	49.37
case 22	5	490.10	607.30	2.07	2.12	44.04	44.47	64.95	3.25	62.92
case 23	21	489.69	893.47	1.66	2.16	44.04	41.62	53.08	11.15	156.21
case 24	6	362.00	862.00	1.66	2.12	44.04	44.47	204.26	12.26	223.89
case 25	10	482.55	571.92	1.30	2.25	44.04	44.47	65.85	6.59	41.81
case 26	20	165.30	206.20	1.66	2.62	44.04	43.23	13.30	2.66	16.36
case 27	8	275.00	440.00	1.66	2.31	44.04	43.18	59.46	4.76	74.55
case 28	24	96.85	230.76	1.66	2.16	44.04	41.62	14.05	3.37	53.39
case 29	16	129.66	258.18	1.66	2.16	44.04	41.62	28.85	4.62	54.53
case 30	24	135.22	290.99	1.66	2.16	44.04	41.62	16.81	4.03	61.56
case 31	16	192.88	329.06	1.66	2.31	44.04	43.18	33.90	5.42	57.33
case 32	12	116.00	209.00	1.04	1.75	44.04	43.18	20.33	2.44	39.17
case 33	8	275.00	411.00	1.66	2.31	44.04	43.18	51.77	4.14	61.66
case 34	8	367.26	586.95	1.66	2.12	44.04	44.47	79.23	6.34	99.27

Case	N_{cr} (g N m^{-2} yr^{-1})	$ANPP_{ck}$ (g m^{-2} yr^{-1})	$ANPP_{max}$ (g m^{-2} yr^{-1})	N content$_{ck}$ (%)	N content (%)	C content$_{ck}$ (%)	C content (%)	NRF (%)	MNRC (g N m^{-2} yr^{-1})	Maximum N-induced C gain (g C m^{-2} yr^{-1})
case 35	5	177.27	215.15	1.76	2.27	44.04	45.90	35.31	1.77	20.69
case 36	11	195.44	406.92	1.66	2.31	44.04	43.18	58.66	6.16	89.66
case 37	10.5	159.00	231.00	1.66	2.31	44.04	43.18	25.69	2.70	29.73
case 38	5	185.00	227.00	1.66	2.01	44.04	45.90	29.98	1.50	22.72
case 39	10	180.00	251.00	1.66	2.12	44.04	44.47	23.29	2.33	32.35
case 40	10	188.80	266.02	1.66	2.12	44.04	44.47	25.01	2.50	35.15
case 41	10	232.50	295.20	1.66	2.31	44.04	42.77	23.94	2.39	28.88
case 42	5	196.00	291.00	1.66	2.12	44.04	44.47	52.12	2.61	47.26
case 43	20	252.82	476.76	2.02	2.16	43.94	41.62	48.41	9.68	91.90
case 44	3	344.30	402.20	1.66	2.01	44.04	45.90	95.27	2.38	32.99
case 45	5	232.44	376.86	1.04	2.12	28.64	44.47	33.12	1.66	52.21
case 46	20	727.64	1225.97	1.66	2.16	44.04	41.62	100.28	20.06	209.62
case 47	20	461.67	667.25	1.66	2.62	44.04	43.23	49.13	9.83	85.18
case 48	30	338.73	467.26	1.66	2.31	44.04	42.77	17.24	5.17	50.67
case 49	20	256.60	366.06	1.66	2.31	44.04	42.77	26.67	5.33	45.27
case 50	20	522.93	709.70	1.66	2.62	44.04	43.23	49.60	9.92	76.55
case 51	10	160.00	332.00	1.66	2.12	44.04	44.47	43.78	4.38	77.17
case 52	10	140.10	214.80	1.66	2.31	44.04	43.18	22.25	2.22	33.82
case 53	9.2	319.00	438.00	1.66	2.12	44.04	44.47	43.30	3.98	54.29
case 54	4.67	167.00	282.00	1.66	2.01	44.04	45.90	62.23	2.91	55.90
case 55	5	216.67	283.33	1.00	1.25	44.04	45.90	27.20	1.36	34.64
case 56	3	202.08	245.83	0.93	1.12	44.04	45.90	35.20	0.88	23.85
case 57	10	323.00	770.00	1.66	2.12	44.04	44.47	109.54	10.95	200.16
case 58	9.17	194.00	386.00	1.66	2.31	44.04	43.18	54.07	4.96	86.21
case 59	3.00	113.00	146.00	1.09	1.33	47.11	46.88	23.60	0.71	15.21
case 60	15	1630.00	3014.00	1.66	2.62	44.04	43.23	283.92	42.59	583.73
case 61	14	872.00	1636.00	0.33	2.16	44.04	41.62	36.71	5.14	322.47
case 62	14	834.00	1330.00	0.33	2.62	44.04	43.23	26.89	3.76	207.06
case 63	14	825.00	1290.00	0.33	2.16	44.04	41.62	25.70	3.60	193.75
case 64	22	616.00	1046.00	0.33	0.64	44.04	41.62	21.19	4.66	164.06
case 65	4	131.00	159.00	1.70	2.12	44.04	44.47	30.87	1.23	15.29
case 66	9	70.00	258.00	1.66	2.31	44.04	43.18	47.84	4.31	83.90
case 67	8	592.89	761.26	1.66	2.12	44.04	44.47	74.81	6.28	77.42
case 68	5	495.00	665.00	1.66	2.12	44.04	44.47	108.71	5.87	77.72
case 69	17	426.70	1085.68	1.66	2.16	44.04	41.62	125.75	21.38	281.49
case 70	17	541.00	980.00	1.66	2.62	44.04	43.23	98.28	16.71	185.46

Case	N_{cr} (g N m^{-2} yr^{-1})	$ANPP_{ck}$ (g m^{-2} yr^{-1})	$ANPP_{max}$ (g m^{-2} yr^{-1})	N $content_{ck}$ (%)	N content (%)	C $content_{ck}$ (%)	C content (%)	NRF (%)	MNRC (g N m^{-2} yr^{-1})	Maximum N-induced C gain (g C m^{-2} yr^{-1})
case 71	17	393.00	712.00	1.66	2.62	44.04	43.23	71.41	12.14	134.77
case 72	61.3	649.00	1424.00	1.66	2.31	44.04	42.77	36.11	22.13	323.21
case 73	35	509.00	1084.00	1.66	2.31	44.04	42.77	47.43	16.60	239.45
case 74	8	44.20	99.00	1.66	2.12	44.04	44.47	17.05	1.36	24.56
case 75	32	474.00	841.00	1.66	2.31	44.04	42.77	36.14	11.56	150.94
case 76	32	561.00	967.00	1.66	2.31	44.04	42.77	40.72	13.03	166.51
case 77	8	60.30	109.70	1.66	2.12	44.04	44.47	16.54	1.32	22.23
case 78	8	68.60	105.00	1.66	2.12	44.04	44.47	13.57	1.09	16.48
case 79	15	1228.00	2226.00	1.66	2.31	44.04	43.18	207.02	31.05	420.47
case 80	20	229.00	318.00	1.25	2.62	44.04	43.23	13.65	2.73	36.64
case 81	53	265.00	815.00	1.66	2.31	44.04	42.77	27.19	14.44	231.86
case 82	30	930.00	1350.00	1.66	2.31	44.04	42.77	52.51	15.75	167.82
case 83	36	485.00	641.00	1.66	2.31	44.04	42.77	18.77	6.76	60.56
case 84	6	55.00	90.00	1.66	2.12	44.04	44.47	16.56	0.99	15.80
case 85	6	56.00	107.00	1.66	2.12	44.04	44.47	22.29	1.34	22.92
case 86	34	1050.00	1595.00	1.66	2.31	44.04	42.77	57.13	19.42	219.75
case 87	47	570.00	1310.00	1.66	2.31	44.04	42.77	43.90	20.81	309.24
case 88	24	345.00	833.00	1.66	2.31	44.04	42.77	51.02	12.25	194.76
case 89	24	911.00	1158.00	1.66	2.16	44.04	41.62	41.06	9.86	80.77

Note:

N_{cr}, critical nitrogen (N) rate;

$ANPP_{ck}$, initial aboveground net primary productivity in Appendix S2: Fig. S2;

$ANPP_{max}$, Maximum aboveground net primary productivity at N_{cr} in Appendix S2: Fig. S2;

N $content_{ck}$, Mean value of N content in control group in Appendix S2: Table S3;

N content, Mean value of N content of the corresponding intervals in Appendix S2: Table S3 according to the N_{cr};

C $content_{ck}$, Mean value of carbon (C) content in control group in Appendix S2: Table S3;

C content, Mean value of C content of the corresponding intervals in Appendix S2: Table S3 according to the N_{cr};

NRF, N retention fraction at N_{cr}, NRF = ($ANPP_{max}$ × N content - $ANPP_{ck}$ × N $content_{ck}$)/N_{cr};

MNRC, Maximum N retention capacity, MNRC = $ANPP_{max}$ × N content - $ANPP_{ck}$ × N $content_{ck}$;

Maximum N-induced C gain, Maximum N-induced C gain = $ANPP_{max}$ × C content - $ANPP_{ck}$ × C $content_{ck}$.

Table S3. Mean values of foliar carbon (C) and nitrogen (N) content grouped according to N addition rate. The reference sources are shown in the Source 2.

N rate (g N m^{-2} yr^{-1})	N content (%)	C content (%)
CK	1.66	44.04
0-5	2.01	45.90
5-10	2.12	44.47
10-15	2.31	43.18
15-20	2.62	43.23
20-25	2.16	41.62
>25	2.31	42.77

Table S4. Simple regression (Pearson) results of the maximum aboveground net primary productivity ($ANPP_{max}$), critical N rate (N_{cr}), N retention fraction (NRF) at N_{cr}, maximum N retention capacity (MNRC) and Maximum N-induced C gain with predictors.

Variable	Predictor	*df*	*F*	*P*	R^2
$ANPP_{max}$	$ANPP_{ck}$	1, 88	2427.58	<0.001	0.97
N_{cr}	MAP	1, 88	239.80	<0.001	0.73
	Soil C:N	1, 80	147.71	<0.001	0.65
NRF	$ANPP_{ck}$	1, 88	103.81	<0.001	0.54
	Soil C:N	2, 78	26.21	<0.001	0.40
MNRC	$ANPP_{ck}$	1, 88	320.11	<0.001	0.78
Maximum N-induced C gain	$ANPP_{ck}$	1, 88	388.59	<0.001	0.82

Note: Predictors are initial ANPP ($ANPP_{ck}$), mean annual precipitation (MAP) and soil C:N ratio.

Table S5 Fencing management influenced on critical N rate (N_{cr}), N retention fraction (NRF) at N_{cr}, maximum N retention capacity (MNRC) and Maximum N-induced C gain. Student's t-tests were performed between fencing and grazing. Bold values indicate significance (P <0.05).

Group	N_{cr} (g N m^{-2} yr^{-1})	NRF (%)	MNRC (g N m^{-2} yr^{-1})	Maximum N-induced C gain (g C m^{-2} yr^{-1})
Fencing	8.06 ± 2.29	43.50 ± 2.63	3.51 ± 2.72	49.12 ± 2.51
Grazing	13.21 ± 2.39	33.61 ± 1.85	4.44 ± 3.05	71.49 ± 3.17
**Significance**	t = -2.69, df = 87	t = 1.54, df = 86.98	t = -1.04, df = 87	t = -1.70, df = 87
	***P* = 0.009**	P = 0.127	P = 0.302	P = 0.093

Table S6 Grassland type influenced on critical N rate (N_{cr}), N retention fraction (NRF) at N_{cr}, maximum N retention capacity (MNRC) and Maximum N-induced C gain. *F* statistics for one-way ANOVAs are provided. The same letters in a column indicate insignificant differences ($P > 0.05$) between grassland types. Bold values indicate significance ($P < 0.05$).

Group	N_{cr} (g N m^{-2} yr^{-1})	NRF (%)	MNRC (g N m^{-2} yr^{-1})	Maximum N-induced C gain (g C m^{-2} yr^{-1})
Meadow	6.19 ± 2.88 [b]	47.19 ± 2.58 [a]	2.92 ± 2.45	39.77 ± 2.32
Steppe	11.65 ± 2.14 [a]	35.20 ± 2.10 [ab]	4.10 ± 2.91	63.68 ± 2.89
Desert steppe	10.00 ± 1.76 [ab]	17.90 ± 3.27 [b]	1.79 ± 2.23	30.85 ± 2.94
**Significance**	$F_{2,81} = 4.72$	$F_{2,81} = 2.73$	$F_{2,81} = 1.94$	$F_{2,81} = 2.54$
	$\boldsymbol{P = 0.012}$	$P = 0.071$	$P = 0.150$	$P = 0.085$

Table S7. Regression results of N retention capacity and N-induced C gain with N addition in Namco alpine steppe and Tibetan Plateau.

	N retention capacity ($g N m^{-2} yr^{-1}$; $Tg N yr^{-1}$)				N-induced C gain ($g C m^{-2} yr^{-1}$; $Tg C yr^{-1}$)			
	df	*F*	*P*	R^2	*df*	*F*	*P*	R^2
Namco alpine steppe	2, 34	89.12	<0.001	0.64	2, 34	90.17	<0.001	0.38
Tibetan Plateau	2, 122	34.53	<0.001	0.40	2, 122	68.18	<0.001	0.28

Table S8. Regression results of N retention capacity and N-induced C gain with N addition in eight global sample sites.

	N retention capacity (g N m^{-2} yr^{-1})				N-induced C gain (g C m^{-2} yr^{-1})			
	df	*F*	*P*	R^2	*df*	*F*	*P*	R^2
Minnesota, USA	2, 6	55.63	<0.001	0.87	2, 6	111.35	<0.001	0.93
Michigan, USA	2, 6	48.78	<0.001	0.70	2, 6	361.19	<0.001	0.94
Colorado, USA	2, 2	1970.64	<0.001	0.99	2, 2	32.45	0.03	0.89
Wageningen, NL	2, 6	1260.23	<0.001	0.98	2, 6	1134.45	<0.001	0.98
Duolun, CN	2, 4	61.58	<0.001	0.88	2, 4	120.63	<0.001	0.93
Namco, CN	2, 4	133.06	<0.001	0.96	2, 4	53.79	0.001	0.90
Gippsland, AU	2, 3	8854.81	<0.001	0.99	2, 3	447.68	<0.001	0.99
Margaret River, AU	2, 4	58.00	0.001	0.88	2, 4	158.52	<0.001	0.95

Note: USA, America; NL, Netherlands; CN, China; AU, Australia.

Table S9. Regression results of the aboveground net primary productivity (ANPP), N retention fraction (NRF), N retention capacity and N-induced C gain with N addition in different areas of the Tibetan Plateau and Inner Mongolia.

	Tibetan Plateau				Inner Mongolia			
	Xizang	Qinghai	Gansu	Sichuan	Xilingol	Hulun Buir	Ordos	Hohhot
ANPP (g m^{-2} yr^{-1})								
df	1, 47	-	1, 27	1, 8	2, 92	2, 8	2, 4	2, 27
F	0.74	-	18.28	2.86	15.91	15.38	46.22	1.39
P	0.39	-	<0.001	0.13	<0.001	0.002	0.002	0.27
R^2	0.02	-	0.40	0.26	0.26	0.79	0.96	0.09
NRF (%)								
df	2, 36	2, 25	2, 19	2, 5	2, 77	-	2, 3	1, 23
F	29.12	27.30	6.13	1.98	35.57	-	27.60	3.48
P	<0.001	<0.001	0.009	0.23	<0.001	-	0.01	0.07
R^2	0.62	0.69	0.39	0.44	0.48	-	0.95	0.13
N retention capacity (g N m^{-2} yr^{-1})								
df	2, 47	2, 34	2, 26	2, 8	2, 93	2, 9	2, 5	2, 28
F	90.84	93.92	24.81	23.29	322.37	156.34	210.91	50.10
P	<0.001	<0.001	<0.001	<0.001	<0.001	<0.001	<0.001	<0.001
R^2	0.48	0.58	0.66	0.66	0.68	0.93	0.94	0.41
N-induced C gain (g C m^{-2} yr^{-1})								
df	2, 47	2, 34	1, 27	2, 8	2, 93	2, 9	2, 5	2, 28
F	55.37	83.46	12.15	12.02	278.33	112.75	66.31	33.98
P	<0.001	<0.001	0.002	0.004	<0.001	<0.001	<0.001	<0.001
R^2	0.34	0.58	0.31	0.47	0.62	0.89	0.79	0.33

Supplementary Figures

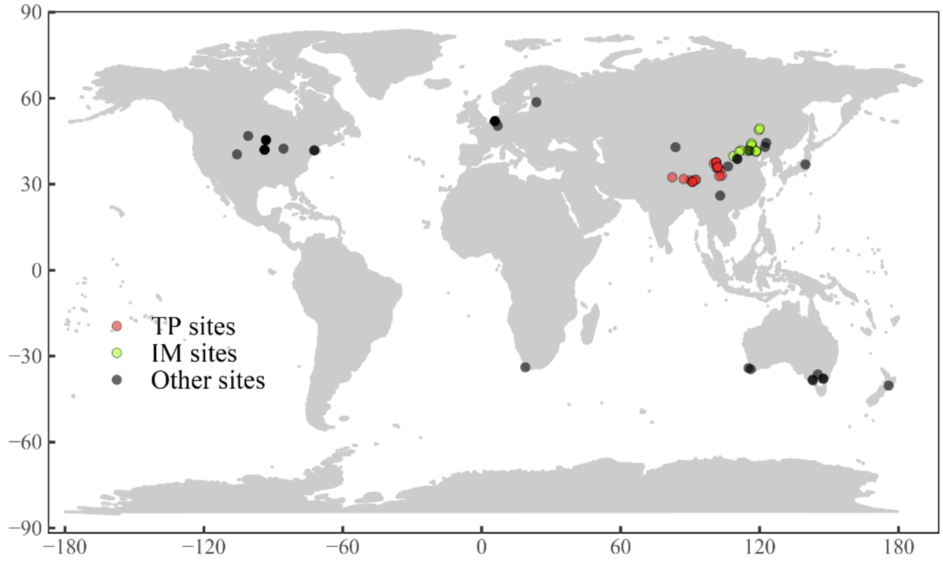

Figure S1. Site distribution maps of 89 N fertilization experiments. Red points indicate experiments were conducted on the Tibetan Plateau (TP) grassland, green points indicate experiments were conducted on the Inner Mongolia (IM) grassland, and black points indicate experiments were conducted on the other terrestrial grassland in the world.

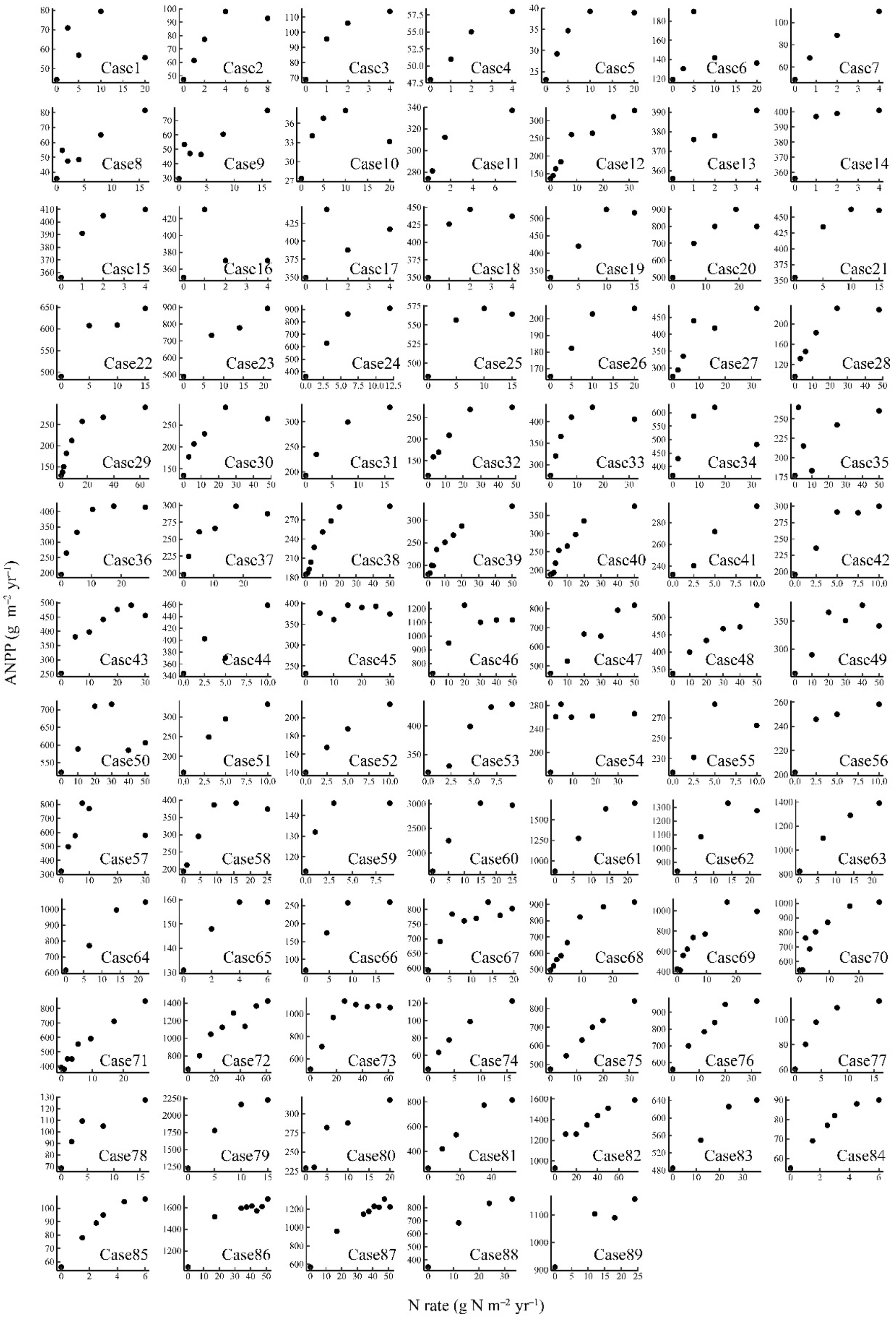

Figure S2. Relationship between aboveground net primary productivity (ANPP) and N addition rate in the 89 cases.

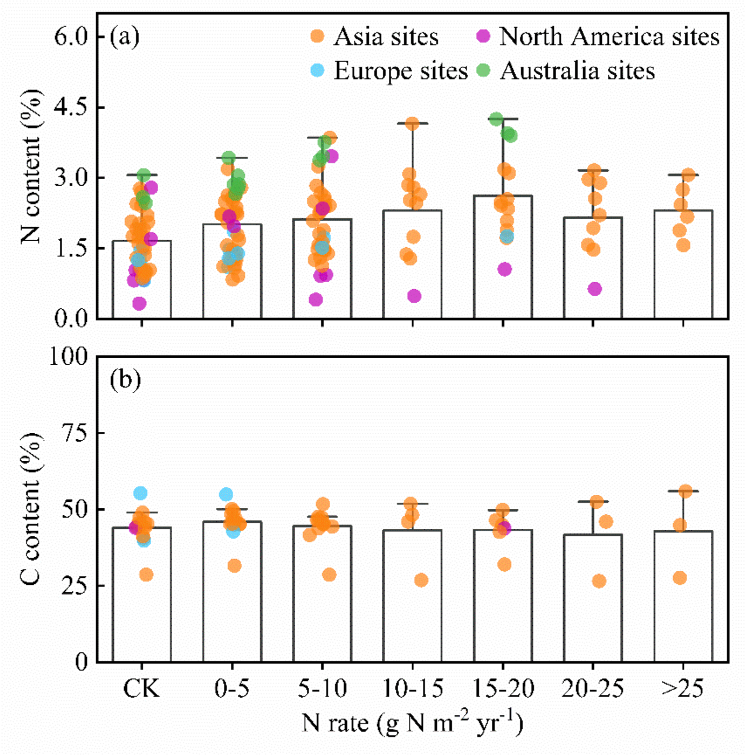

Figure S3. Response of global foliar N and C content to N addition rate. Data are shown as mean ± SD.

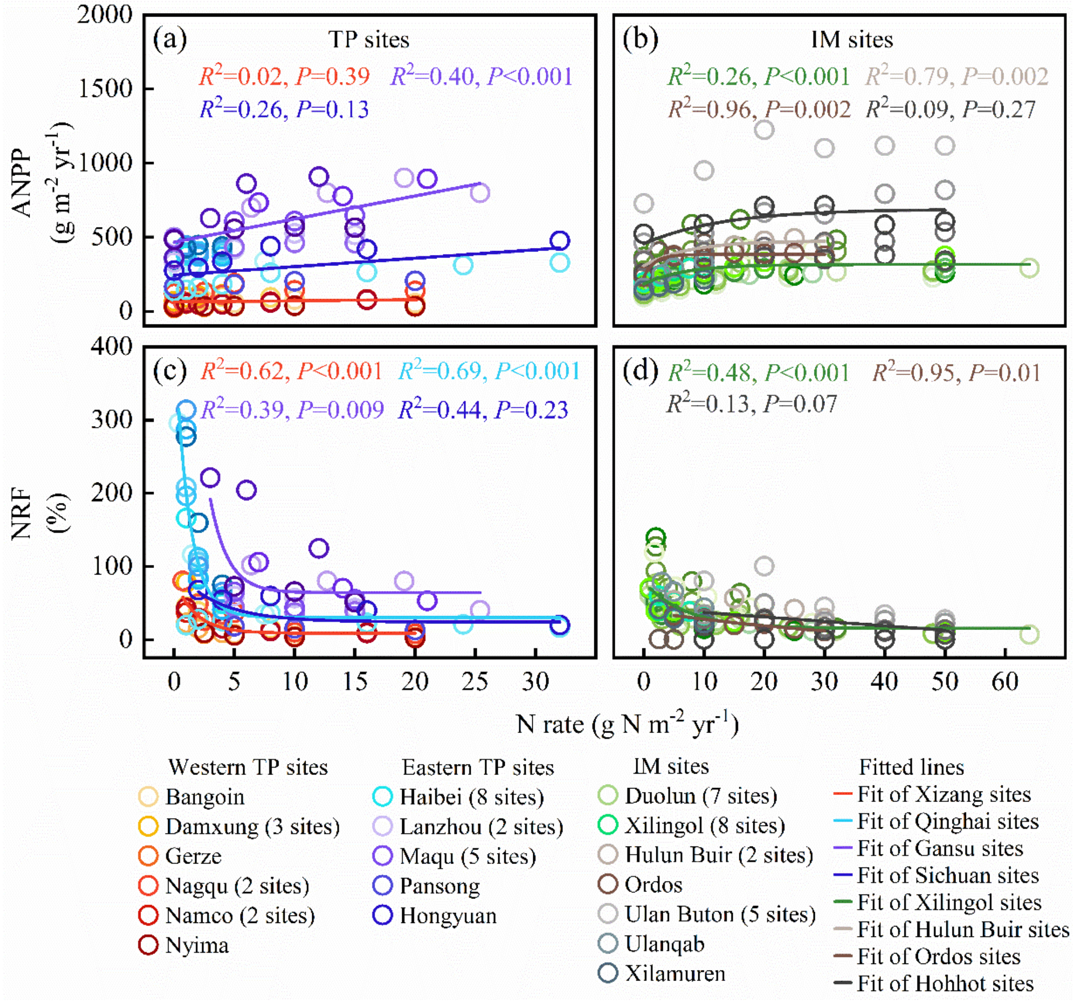

Figure S4. The response of aboveground net primary productivity (ANPP) and N retention fraction (NRF) to N addition rate in Tibetan Plateau (TP) alpine grassland and Inner Mongolia (IM) grassland. The left panels represent TP sample plots and the right panels represent IM sample plots. Simple regression analyses were implemented separately for the western TP (Xizang, n = 49), eastern TP (Qinghai, n = 36; Gansu, n = 29; and Sichuan, n = 10) and four regions in IM (Xilingol, n = 95; Hulun Buir, n = 11; Ordos, n = 7; and Hohhot, n = 30). Full regression results are shown in Appendix S2: Table S9. Points of each color represent a sampling plot and fitting lines of each color represent a region.

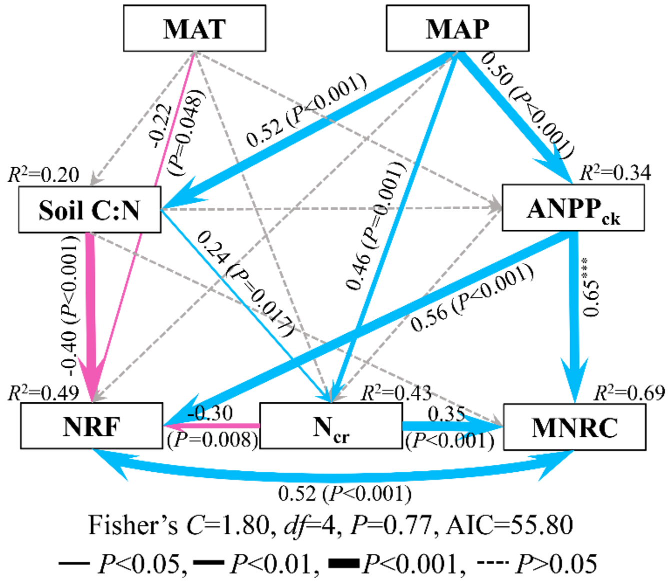

Figure S5. Structural equation modeling describes the influence of environmental factors and initial aboveground net primary productivity ($ANPP_{ck}$) on critical N rate (N_{cr}), N retention fraction (NRF) at N_{cr} and maximum N retention capacity (MNRC). Blue arrows indicate positive correlations between predictor and response variables, red arrows indicate negative correlations, and dashed arrows indicate no significant relationships between variables. The width of the arrows indicates the strength of the relationship. Numbers on the arrow indicates the path coefficient. Fishec's *C*, model degree of freedom (*df*), *P*-value and Akaike information criteria (AIC) of the model are shown. The explained variance of all predictors on the response variables are denoted by R^2.

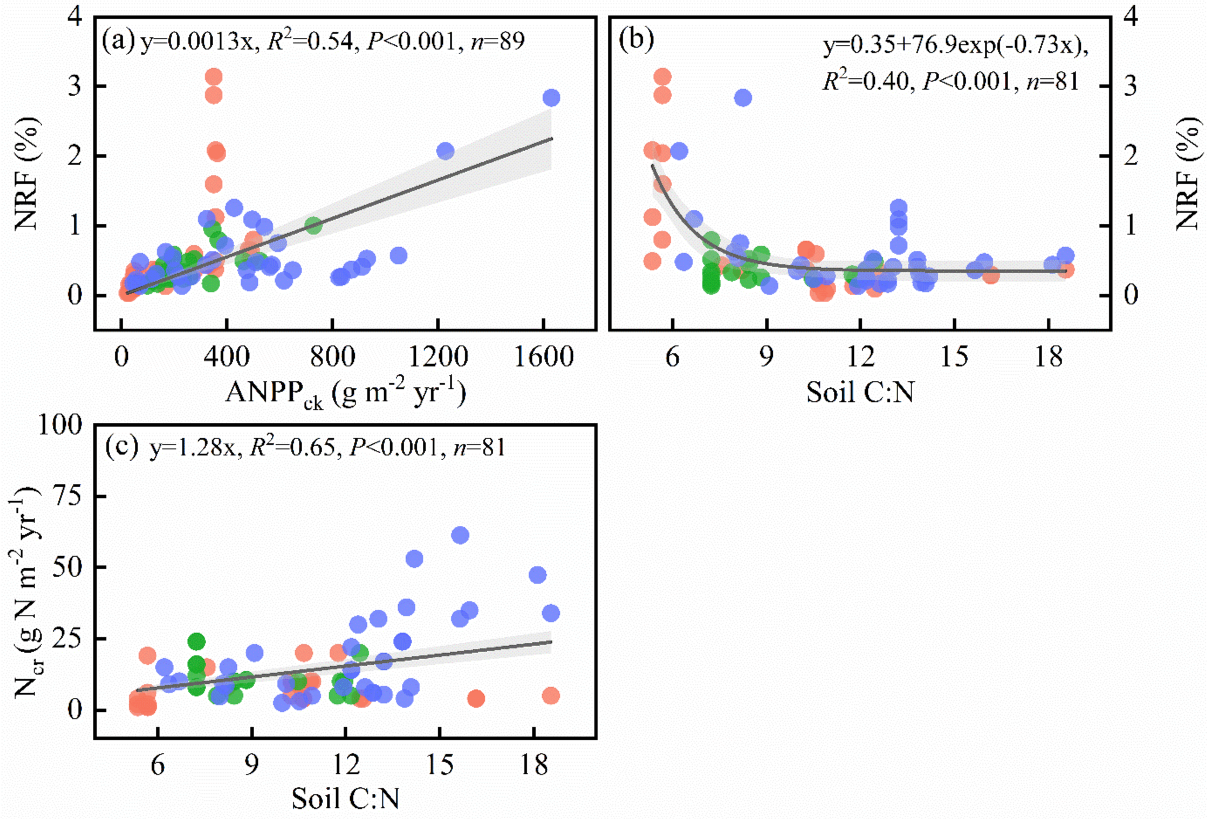

Figure S6. The relationship between (a) N retention fraction (NRF) at critical N rate (N_{cr}) and initial aboveground net primary productivity ($ANPP_{ck}$), (b) NRF at N_{cr} and soil C:N, and (c) N_{cr} and soil C:N. The red points represent Tibetan Plateau (TP) sampling plots, green points represent Inner Mongolia (IM) sampling plots, and blue points represent other global grassland sampling plots. Simple regression analyses were implemented. Full regression results are shown in Appendix S2: Table S4. The gray areas indicate the 95% confidence interval.

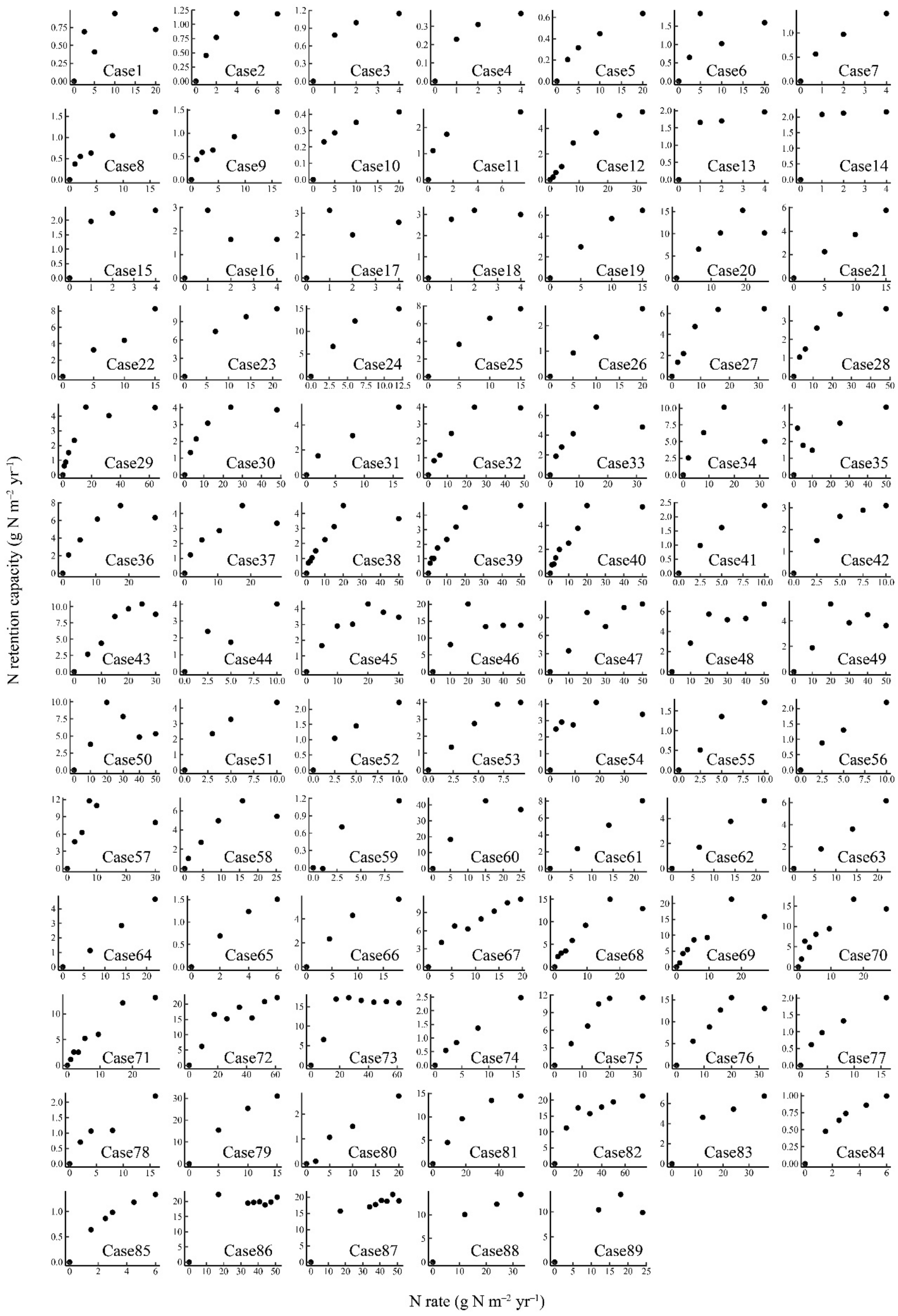

Figure S7. Relationship between N retention capacity and N addition rate in the 89 cases.

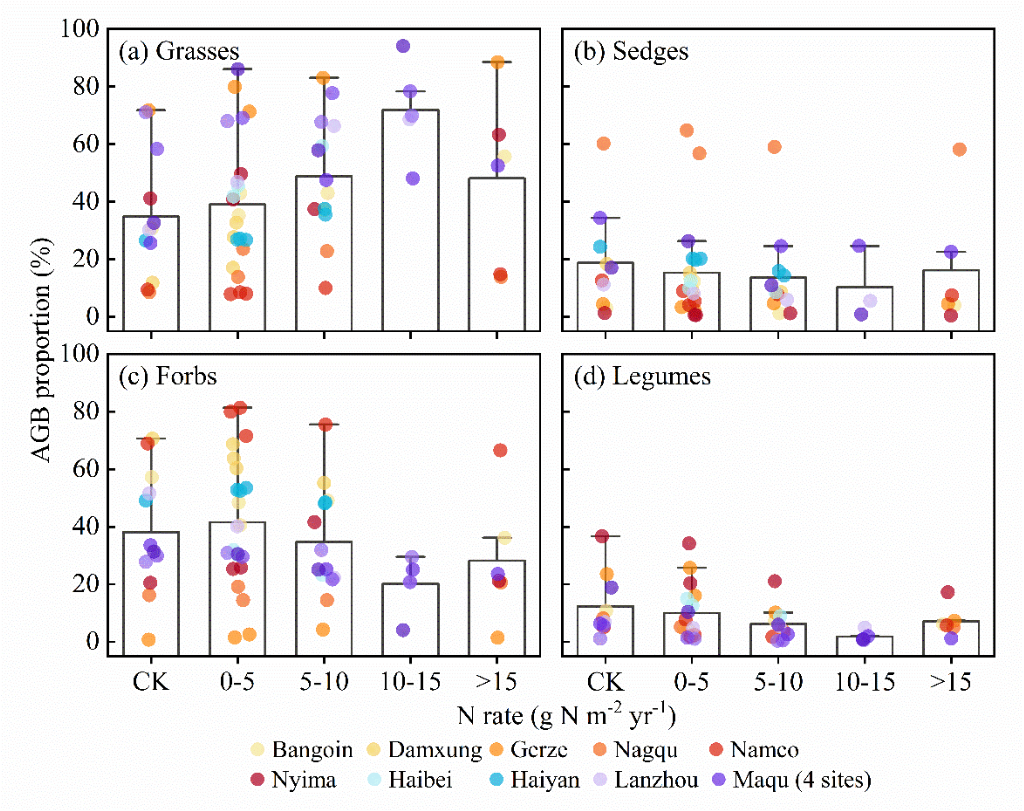

Figure S8. Aboveground biomass (AGB) proportion of four functional groups in Tibetan Plateau grasslands in response to N addition. (a) Grasses, (b) Sedges. (c) Forbs. (d) Legumes. We selected 13 cases from the data synthesis on the Tibetan Plateau and each case included at least three functional groups. Data are shown as mean ± SD.

References Sources

Source 1: References for the 89 experimental cases used to assess and compare nitrogen limitation on the Tibetan Plateau.

Anger M, Hoffmann C, Kühbauch W (2003) Nitrous oxide emissions from artificial urine patches applied to different N-fertilized swards and estimated annual N_2O emissions for differently fertilized pastures in an upland location in Germany. Soil Use Manage 19: 104-111. https://doi.org/10.1111/j.1475-2743.2003.tb00288.x

Bai YF, Wu JG, Clark CM, Naeem S, Pan QM, Huang JH, Zhang LX, Han XG (2010) Tradeoffs and thresholds in the effects of nitrogen addition on biodiversity and ecosystem functioning: evidence from inner Mongolia Grasslands. Glob Change Biol 16: 358-372. https://doi.org/10.1111/j.1365-2486.2009.01950.x

Bolland MDA, Guthridge IF (2007) Responses of intensively grazed dairy pastures to applications of fertiliser nitrogen in south-western Australia. Aust J Exp Agr 47: 927-941. https://doi.org/10.1071/ea06014

Bowman WD, Gartner JR, Holland K, Wiedermann M (2006) Nitrogen critical loads for alpine vegetation and terrestrial ecosystem response: Are we there yet? Ecol Appl 16: 1183-1193. https://doi.org/10.1890/1051-0761(2006)016[1183:NCLFAV]2.0.CO;2

Chen WN, Chen FJ (2017) Response of biomass and species diversity to nitrogen addition in alpine meadows. Pratacult Sci 34: 1082-1089. https://doi.org/10.11829/j.issn.1001-0629.2016-0429 (in Chinese with English abstract)

Chen WQ, Zhang YJ, Mai XH, Shen Y (2016) Multiple mechanisms contributed to the reduced stability of Inner Mongolia grassland ecosystem following nitrogen enrichment. Plant Soil 409: 283-296. https://doi.org/10.1007/s11104-016-2967-1

Collins SA, Allinson DW (2004) Soil nitrate concentrations used to predict nitrogen sufficiency in relation to yield in perennial grasslands. Agron J 96: 1272-1281. https://doi.org/10.2134/agronj2004.1272

Fang HJ, Cheng SL, Yu GR, Cooch J, Wang YS, Xu MJ, Li LS, Dang XS, Li YN (2014) Low-level nitrogen deposition significantly inhibits methane uptake from an alpine meadow soil on the Qinghai-Tibetan Plateau. Geoderma 213: 444-452. https://doi.org/10.1016/j.geoderma.2013.08.006

Fang Y, Xun F, Bai WM, Zhang WH, Li LH (2012) Long-term nitrogen addition leads to loss of species richness due to litter accumulation and soil acidification in a temperate steppe. Plos One 7: e47369. https://doi.org/10.1371/journal.pone.0047369

Gao HY, Hong M, Huo LX, Liu PF, Chang F (2018) Effect of water and nitrogen interaction on plant species diversity and biomass in a desert grassland. Pratacult Sci 35: 36-45. https://doi.org/10.11829/j.issn.1001-0629.2017-0167. (in Chinese with English abstract)

Gourley CJP, Hannah MC, Chia KTH (2017) Predicting pasture yield response to nitrogenous fertiliser in Australia using a meta-analysis-derived model, with field validation. Soil Res 55: 567-578. https://doi.org/10.1071/sr17032

Han XW, Tsunekawa A, Tsubo M, Li SQ (2011) Aboveground biomass response to increasing nitrogen deposition on grassland on the northern Loess Plateau of China. Acta Agr Scand B-S P 61: 112-121. https://doi.org/10.1080/09064710903544201

Hao TX, Song L, Goulding K, Zhang FS, Liu XJ (2017) Cumulative and partially recoverable impacts of nitrogen addition on a temperate steppe. Ecol Appl 28: 237–248. https://doi.org/10.1002/eap.1647

He D, Li XL, He F, Wan LQ, Li CR (2009) Effect of nitrogen fertilizer on biomass and the important values of the

main species in degraded grassland. Chinese J Grassl 31: 42-46. (in Chinese with English abstract)

He X, Ma WH, Liang CZ, Hong M, Chai X, Zhao B, Zhang Y, Yang SH, Zhang JX, Xin XP (2015) Effects of nutrient additions on community biomass varied among different grassland ecosystems of Inner Mongolia. Acta Sci Naturalium Univ Pekinensis 51: 657-666. https://doi.org/10.13209/j.0479-8023.2015.090. (in Chinese with English abstract)

Heggenstaller AH, Moore KJ, Liebman M, Anex RP (2009) Nitrogen influences biomass and nutrient partitioning by perennial, warm-season grasses. Agron J 101: 1363-1371. https://doi.org/10.2134/agronj2008.0225x

Hoogendoorn CJ, Lambert MG, Devantier BP, Theobald PW, Park ZA (2017) Nitrogen fertiliser application rates and nitrogen leaching in intensively managed sheep grazed hill country pastures in New Zealand. N Z J Agric Res 60: 154-172. https://doi.org/10.1080/00288233.2017.1287100

Labuschagne J, Hardy MB, Agenbag GA (2013) The effects of strategic nitrogen fertiliser application during the cool season on perennial ryegrass-white clover pastures in the Western Cape Province 2. Dry matter production. S Afr J Plant Soil 23: 262-268. https://doi.org/10.1080/02571862.2006.10634764

Li DD (2020) Effects of nitrogen and phosphorus addition on community and stoichiometric characteristics of carbon, nitrogen and phosphorus in grassland ecosystem of northern China. Univ Chinese Acad Sci. https://doi.org/10.27558/d.cnki.gsthc.2020.000031. (in Chinese with English abstract)

Li J, Zhang C, Yang Z, Guo H, Zhou X, Du G (2017) Grazing and fertilization influence plant species richness via direct and indirect pathways in an alpine meadow of the eastern Tibetan Plateau. Grass Forage Sci 72: 343-354. https://doi.org/10.1111/gfs.12232

Li KH, Liu XJ, Song L, Gong YM, Lu CF, Yue P, Tian CY, Zhang FS (2015) Response of alpine grassland to

elevated nitrogen deposition and water supply in China. Oecologia 177: 65-72. https://doi.org/10.1007/s00442-014-3122-4

Li LJ, Zeng DH, Yu ZY, Ai GY, Yang D, Mao R (2009) Effects of nitrogen addition on grassland species diversity and productivity in Keerqin Sandy Land. Chinese J Appl Ecol 20: 1838-1844. (in Chinese with English abstract)

Liu WX, Jiang L, Hu SJ, Li LH, Liu LL, Wan SQ (2014) Decoupling of soil microbes and plants with increasing anthropogenic nitrogen inputs in a temperate steppe. Soil Biol Biochem 72: 116-122. https://doi.org/10.1016/j.soilbio.2014.01.022

Liu YJ, Shi GX, Mao L, Cheng G, Jiang SJ, Ma XJ, An LZ, Du GZ, Collins Johnson N, Feng HY (2012) Direct and indirect influences of 8 yr of nitrogen and phosphorus fertilization on *Glomeromycota* in an alpine meadow ecosystem. New Phytol 194: 523-535. https://doi.org/10.1111/j.1469-8137.2012.04050.x

Liu YW, Xu-Ri, Xu XL, Wei D, Wang YH, Wang YS (2013) Plant and soil responses of an alpine steppe on the Tibetan Plateau to multi-level nitrogen addition. Plant Soil 373: 515-529. https://doi.org/10.1007/s11104-013-1814-x

Lorenz RJ, Rogler GA (1972) Forage production and botanical composition of mixed prairie as influenced by nitrogen and phosphorus fertilization. Agron J 64: 244-249. https://doi.org/10.2134/agronj1972.00021962006400020034x

McKenzie FR, Ryan MJ, Jacobs JL, Kearney G (1999) Effect of rate and time of nitrogen application from autumn to midwinter on perennial ryegrass - white clover dairy pastures in western Victoria. 1. Growth and composition. Aust J Agric Res 50: 1059-1065. https://doi.org/10.1071/ar98196

Mori A, Hojito M (2011) Nitrous oxide and methane emissions from grassland treated with bark- or sawdust-containing manure at different rates. Soil Sci Plant Nutr 57: 138-149. https://doi.org/10.1080/00380768.2010.548310

Niinemets Ü, Kull K (2005) Co-limitation of plant primary productivity by nitrogen and phosphorus in a species-rich wooded meadow on calcareous soils. Acta Oecol 28: 345-356. https://doi.org/10.1016/j.actao.2005.06.003

Peng YF, Li F, Zhou GY, Fang K, Zhang DY, Li CB, Yang GB, Wang GQ, Wang J, Yang YH (2017) Linkages of plant stoichiometry to ecosystem production and carbon fluxes with increasing nitrogen inputs in an alpine steppe. Glob Change Biol 23: 5249-5259. https://doi.org/10.1111/gcb.13789

Qiu B, Luo YJ (2004) Effects of fertilizer gradients on productivity and species diversity in a degraded alpine meadow. J Lanzhou Univ Nat Sci 40: 56-59. https://doi.org/10.13885/j.issn.0455-2059.2004.03.014. (in Chinese with English abstract)

Ruan LL, Bhardwaj AK, Hamilton SK, Robertson GP (2016) Nitrogen fertilization challenges the climate benefit of cellulosic biofuels. Environ Res Lett 11: 064007. https://doi.org/10.1088/1748-9326/11/6/064007

Schils RLM, van Groenigen JW, Velthof GL, Kuikman PJ (2008) Nitrous oxide emissions from multiple combined applications of fertiliser and cattle slurry to grassland. Plant Soil 310: 89-101. https://doi.org/10.1007/s11104-008-9632-2

Schröder JJ, Assinck FBT, Uenk D, Velthof GL (2010) Nitrate leaching from cut grassland as affected by the substitution of slurry with nitrogen mineral fertilizer on two soil types. Grass Forage Sci 65: 49-57. https://doi.org/10.1111/j.1365-2494.2009.00719.x

Sheng ZL, Huang YM, He KJ, Narigele B, Yang HY, Chen HY, Li EG, Xu X, Duan L (2019) Responses of plant ^{15}N

natural abundance and isotopic fractionation to N addition reflect the N status of a temperate steppe in China. J Plant Ecol 12: 550–563. https://doi.org/10.1093/jpe/rty047

Song B, Sun J, Zhou QP, Zong N, Li LH, Niu SL (2017) Initial shifts in nitrogen impact on ecosystem carbon fluxes in an alpine meadow: patterns and causes. Biogeosciences 14: 3947-3956. https://doi.org/10.5194/bg-14-3947-2017

Song L, Bao X, Liu X, Zhang Y, Christie P, Fangmeier A, Zhang F (2011) Nitrogen enrichment enhances the dominance of grasses over forbs in a temperate steppe ecosystem. Biogeosciences 8: 2341-2350. https://doi.org/10.5194/bg-8-2341-2011

Song L, Bao XM, Liu XJ, Zhang FS (2012a) Impact of nitrogen addition on plant community in a semi-arid temperate steppe in China. J Arid Land 4: 3-10. https://doi.org/10.3724/SP.J.1227.2012.00003

Song MH, Yu FH, Ouyang H, Cao GM, Xu XL, Cornelissen JHC (2012b) Different inter-annual responses to availability and form of nitrogen explain species coexistence in an alpine meadow community after release from grazing. Glob Change Biol 18: 3100-3111. https://doi.org/10.1111/j.1365-2486.2012.02738.x

Sun S, Xing F, Zhao H, Gao Y, Bai Z, Dong Y (2014) Response of bacterial community to simulated nitrogen deposition in soils and a unique relationship between plant species and soil bacteria in the Songnen grassland in Northeastern China. J Soil Sci Plant Nutr 14: 565-580. https://doi.org/10.4067/S0718-95162014005000045

Sun XM, Yu KL, Shugart HM, Wang G (2015) Species richness loss after nutrient addition as affected by N:C ratios and phytohormone GA_3 contents in an alpine meadow community. J Plant Ecol 9: 201-211. https://doi.org/10.1093/jpe/rtv037

Tian QY, Liu NN, Bai WM, Li LH, Chen JQ, Reich PB, Yu Q, Guo DL, Smith MD, Knapp AK, Cheng WX, Lu P,

Gao Y, Yang A, Wang TZ, Li X, Wang ZW, Ma YB, Han XG, Zhang WH (2016) A novel soil manganese mechanism drives plant species loss with increased nitrogen deposition in a temperate steppe. Ecology 97: 65-74. https://doi.org/10.1890/15-0917.1

Tilman D (1987) Secondary succession and the pattern of plant dominance along experimental nitrogen gradients. Ecol Monogr 57: 189-214. https://doi.org/10.2307/2937080

Tilman D (1993) Species richness of experimental productivity gradients: How important is colonization limitation? Ecology 74: 2179-2191. https://doi.org/10.2307/1939572

Wang J, Gao YZ, Zhang YH, Yang JJ, Smith MD, Knapp AK, Eissenstat DM, Han XG (2019) Asymmetry in above- and belowground productivity responses to N addition in a semi-arid temperate steppe. Glob Change Biol 25: 2958-2969. https://doi.org/10.1111/gcb.14719

Xu DH, Fang XW, Zhang RY, Gao TP, Bu HY, Du GZ (2015a) Influences of nitrogen, phosphorus and silicon addition on plant productivity and species richness in an alpine meadow. AoB Plants 7: plv125. https://doi.org/10.1093/aobpla/plv125

Xu XT, Liu HY, Song ZL, Wang W, Hu GZ, Qi ZH (2015b) Response of aboveground biomass and diversity to nitrogen addition along a degradation gradient in the Inner Mongolian steppe, China. Sci Rep 5: 10284. https://doi.org/10.1038/srep10284

Yan YL, Ganjurjav H, Hu GZ, Liang Y, Li Y, He SC, Danjiu LB, Yang J, Gao QZ (2018) Nitrogen deposition induced significant increase of N_2O emissions in an dry alpine meadow on the central Qinghai-Tibetan Plateau. Agr Ecosyst Environ 265: 45-53. https://doi.org/10.1016/j.agee.2018.05.031

Zhang CH (2014) Effects of grazing and fertilization on community productivity and species richness in eastern

alpine meadow of Tibetan plateau. Pratacult Sci 31: 2293-2300. https://doi.org/10.11829\j.issn.1001-0629.2014-0392. (in Chinese with English abstract)

Zhang YD, Shen YX, Liu WY (2004) Fertilization effects of N, P on a grass community at the dry-valley of Jinsha River. Bull Bot Res 24: 59-64. (in Chinese with English abstract)

Zhang YH, Feng JC, Isbell F, Lü XT, Han XG (2015) Productivity depends more on the rate than the frequency of N addition in a temperate grassland. Sci Rep 5: 12558. https://doi.org/10.1038/srep12558

Zhao J, Li W, Jing GH, Wei L, Cheng JM (2017) Responses of species diversity and aboveground biomass to nitrogen addition in fenced and grazed grassland on the Loess Plateau. Acta Prataculturae Sinica 26: 54-64. https://doi.org/10.11686/cyxb2017064 (in Chinese with English abstract)

Zheng Z, Bai WM, Zhang WH (2019) Root trait-mediated belowground competition and community composition of a temperate steppe under nitrogen enrichment. Plant Soil 437: 341–354. https://doi.org/10.1007/s11104-019-03989-z

Zhou XL, Guo Z, Zhang PF, Li HL, Chu CJ, Li XL, Du GZ (2017) Different categories of biodiversity explain productivity variation after fertilization in a Tibetan alpine meadow community. Ecol Evol 7: 3464-3474. https://doi.org/10.1002/ece3.2723

Zhu TH, Cheng SL, Fang HJ, Yu GR, Zheng JJ, Li YN (2011) Early response of soil CO_2 emission to simulating atmospheric nitrogen deposition in an alpine meadow on the Qinghai Tibetan Plateau. Acta Ecol Sin 31: 2687-2696. (in Chinese with English abstract)

Zong N, Shi PL, Song MH, Lin L, Ma WL, Jiang J, Fu G, He YT, Zhang XZ (2012) Clipping alters the response of biomass allocation pattern under nitrogen addition in an alpine meadow on the Tibetan Plateau. J Nat Reso 27:

1696-1707. https://doi.org/10.11849/zrzyxb.2012.10.008

Zong N, Shi PL, Song MH, Zhang XZ, Jiang J, Chai X (2016) Nitrogen critical loads for an alpine meadow ecosystem on the Tibetan Plateau. Environ Manage 57: 531-542. https://doi.org/10.1007/s00267-015-0626-6

Zong N, Song MH, Shi PL, Jiang J, Zhang XZ, Shen ZX (2014) Timing patterns of nitrogen application alter plant production and CO_2 efflux in an alpine meadow on the Tibetan Plateau, China. Pedobiologia 57: 263-269. https://doi.org/10.1016/j.pedobi.2014.08.001

Zong N, Zhao GS, Shi PL (2019) Different sensitivity and threshold in response to nitrogen addition in four alpine grasslands along a precipitation transect on the northern Tibetan Plateau. Ecol Evol 9: 9782-9793. https://doi.org/10.1002/ece3.5514

Source 2: References used to assess the response of foliar carbon (C) and nitrogen (N) content to N fertilization rate.

An Z, Niu DC, Wen HY, Yang Y, Zhang HR, Fu H (2011) Effects of N addition on nutrient resorption efficiency and C:N:P stoichiometric characteristics in *Stipa bungeana* of steppe grasslands in the Loess Plateau, China. Chinese J Plant Ecol 35: 801-807. https://doi.org/10.3724/sp.J.1258.2011.00801. (in Chinese with English abstract)

Bao YJ (2016) Effects of nitrogen addition on ecosystem stoichiomtry characteristics in degraded grasslands. Inner Mongolia Univ. (in Chinese with English abstract)

Bin ZJ, Wang JJ, Zhang WP, Xu DH, Cheng XH, Li KJ, Cao DH (2014) Effects of N addition on ecological stoichiometric characteristics in six dominant plant species of alpine meadow on the Qinghai-Xizang Plateau, China. Chinese J Plant Ecol 38: 231-237. https://doi.org/10.3724/sp.J.1258.2014.00020. (in Chinese with English abstract)

Bobbink R (1991) Effects of nutrient enrichment in dutch chalk grassland. J Appl Ecol 28: 28-41. https://doi.org/10.2307/2404111

Bowman WD, Gartner JR, Holland K, Wiedermann M (2006) Nitrogen critical loads for alpine vegetation and terrestrial ecosystem response: Are we there yet? Ecol Appl 16: 1183-1193. https://doi.org/10.1890/1051-0761(2006)016[1183:NCLFAV]2.0.CO;2

Craine JM, Morrow C, Stock WD (2008) Nutrient concentration ratios and co-limitation in South African grasslands. New Phytol 179: 829-836. https://doi.org/10.1111/j.1469-8137.2008.02513.x

Falk K, Friedrich U, Oheimb GV, Mischke K, Merkle K, Meyer H, Härdtle W (2010) Molinia caerulea responses to

N and P fertilisation in a dry heathland ecosystem (NW-Germany). Plant Ecol: 47-56. https://doi.org/10.1007/s11258-010-9720-2

Gao ZB, Wang HY, Lü XT, Wang ZW (2017) Effects of nitrogen and phosphorus addition on C：N：P stoichiometry in roots and leaves of four dominant plant species in a meadow steppe of Hulunbuir. Chinese J Ecol 36: 80-88. https://doi.org/10.13292/j.1000-4890.201701.015. (in Chinese with English abstract)

Gourley CJP, Hannah MC, Chia KTH (2017) Predicting pasture yield response to nitrogenous fertiliser in Australia using a meta-analysis-derived model, with field validation. Soil Res 55: 567-578. https://doi.org/10.1071/sr17032

Han XW, Tsunekawa A, Tsubo M, Li SQ (2011) Aboveground biomass response to increasing nitrogen deposition on grassland on the northern Loess Plateau of China. Acta Agr Scand B-S P 61: 112-121. https://doi.org/10.1080/09064710903544201

Heggenstaller AH, Moore KJ, Liebman M, Anex RP (2009) Nitrogen influences biomass and nutrient partitioning by perennial, warm-season grasses. Agron J 101: 1363-1371. https://doi.org/10.2134/agronj2008.0225x

Heijmans MMPD, Berendse F, Arp WJ, Masselink AK, Klees H, Visser WD, Breemen NV (2001) Effects of elevated carbon dioxide and increased nitrogen deposition on bog vegetation in the Netherlands. J Ecol: 268-279. https://doi.org/10.1046/j.1365-2745.2001.00547.x

Leith ID, Hicks WK, Fowler D, Woodin SJ (1999) Differential responses of UK upland plants to nitrogen deposition. New Phytol 141: 277-289. https://doi.org/10.1046/j.1469-8137.1999.00333.x

Li DD (2020) Effects of nitrogen and phosphorus addition on community and stoichiometric characteristics of carbon, nitrogen and phosphorus in grassland ecosystem of northern China. Univ Chinese Acad Sci.

https://doi.org/10.27558/d.cnki.gsthc.2020.000031. (in Chinese with English abstract)

Li J, Zhang C, Yang Z, Guo H, Zhou X, Du G (2017a) Grazing and fertilization influence plant species richness via direct and indirect pathways in an alpine meadow of the eastern Tibetan Plateau. Grass Forage Sci 72: 343-354. https://doi.org/10.1111/gfs.12232

Li KH, Liu XJ, Song L, Gong YM, Lu CF, Yue P, Tian CY, Zhang FS (2015) Response of alpine grassland to elevated nitrogen deposition and water supply in China. Oecologia 177: 65-72. https://doi.org/10.1007/s00442-014-3122-4

Li L, Li XY, Liu B, Lei JQ, Yue ZW, Li CD (2020) Imbalanced stoichiometric patterns in foliar nutrient resorption response to N and P addition in grazing alpine grassland. Acta Oecol 102: 103505. https://doi.org/10.1016/j.actao.2019.103505

Li Y, Lin L, Zhu WY, Zhang ZH, He JS (2017b) Responses of leaf traits to nitrogen and phosphorus additions across common species in an alpine grassland on the Qinghai-Tibetan Plateau. Acta Sci Naturalium Univ Pekinensis 53. http://www.cnki.net/kcms/doi/10.13209/j.0479-8023.2017.057.html

Litaor MI, Seastedt TR, Sackett LC (2008) Nutrient status in alpine soils of the Colorado front range using the nitrogen/phosphorus ratio index. Soil Sci Soc Am J 72: 1628-1636. https://doi.org/10.2136/sssaj2006.0434

Liu HM, Li J, Huang-Fu CH, Chen XW, Yang DL (2016) Effects of long-term nitrogen addition on photosynthetic characteristics and leaf traits of *Stipa baicalensis* in Inner Mongolia, China. Acta Prataculturae Sinica 25: 76-85. https://doi.org/10.11686/cyxb2016110

Liu YW, Xu-Ri, Xu XL, Wei D, Wang YH, Wang YS (2013) Plant and soil responses of an alpine steppe on the Tibetan Plateau to multi-level nitrogen addition. Plant Soil 373: 515-529. https://doi.org/10.1007/s11104-013-

1814-x

Long M, Wu HH, Smith MD, La Pierre KJ, Lu XT, Zhang HY, Han XG, Yu Q (2016) Nitrogen deposition promotes phosphorus uptake of plants in a semi-arid temperate grassland. Plant Soil 408: 475-484. https://doi.org/10.1007/s11104-016-3022-y

Lü XT, Han XG (2009) Nutrient resorption responses to water and nitrogen amendment in semi-arid grassland of Inner Mongolia, China. Plant Soil 327: 481-491. https://doi.org/10.1007/s11104-009-0078-y

Lü XT, Hu YY, Zhang HY, Wei H, Hou SL, Yang GJ, Liu ZY, Wang XB (2018) Intraspecific variation drives community-level stoichiometric responses to nitrogen and water enrichment in a temperate steppe. Plant Soil 423: 307-315. https://doi.org/10.1007/s11104-017-3519-z

Lü XT, Kong DL, Pan QM, Simmons ME, Han XG (2012) Nitrogen and water availability interact to affect leaf stoichiometry in a semi-arid grassland. Oecologia 168: 301-310. https://doi.org/10.1007/s00442-011-2097-7

Lü XT, Reed SC, Yu Q, Han XG (2015) Nutrient resorption helps drive intra-specific coupling of foliar nitrogen and phosphorus under nutrient-enriched conditions. Plant Soil 398: 111-120. https://doi.org/10.1007/s11104-015-2642-y

Mao R, Zhang XH, Song CC (2014) Effects of nitrogen addition on plant functional traits in freshwater wetland of Sanjiang Plain, Northeast China. Chinese Geogr Sci 24: 674-681. https://doi.org/10.1007/s11769-014-0691-4

Niinemets Ü, Kull K (2005) Co-limitation of plant primary productivity by nitrogen and phosphorus in a species-rich wooded meadow on calcareous soils. Acta Oecol 28: 345-356. https://doi.org/10.1016/j.actao.2005.06.003

Peng YF, Li F, Zhou GY, Fang K, Zhang DY, Li CB, Yang GB, Wang GQ, Wang J, Yang YH (2017) Linkages of plant stoichiometry to ecosystem production and carbon fluxes with increasing nitrogen inputs in an alpine

steppe. Glob Change Biol 23: 5249-5259. https://doi.org/10.1111/gcb.13789

Rejmánková E, Snyder JM (2008) Emergent macrophytes in phosphorus limited marshes: do phosphorus usage strategies change after nutrient addition? Plant Soil 313: 141-153. https://doi.org/10.1007/s11104-008-9687-0

Saneoka H, Moghaieb REA, Premachandra GS, Fujita K (2004) Nitrogen nutrition and water stress effects on cell membrane stability and leaf water relations in *Agrostis palustris* Huds. Environ Exp Bot 52: 131-138. https://doi.org/10.1016/j.envexpbot.2004.01.011

Sheng ZL, Huang YM, He KJ, Narigele B, Yang HY, Chen HY, Li EG, Xu X, Duan L (2019) Responses of plant ^{15}N natural abundance and isotopic fractionation to N addition reflect the N status of a temperate steppe in China. J Plant Ecol 12: 550–563. https://doi.org/10.1093/jpe/rty047

Song L, Bao X, Liu X, Zhang Y, Christie P, Fangmeier A, Zhang F (2011) Nitrogen enrichment enhances the dominance of grasses over forbs in a temperate steppe ecosystem. Biogeosciences 8: 2341-2350. https://doi.org/10.5194/bg-8-2341-2011

Sparrius LB, Kooijman AM, Sevink J (2012) Response of inland dune vegetation to increased nitrogen and phosphorus levels. Appl Veg Sci: 40-50. https://doi.org/10.1111/j.1654-109X.2012.01206.x

Vitousek PM (1998) Foliar and litter nutrients, nutrient resorption, and decomposition in Hawaiian *Metrosideros polymorpha*. Ecosystems 1: 401-407. https://doi.org/10.1007/s100219900033

Vitousek PM, Walker LR, Whiteaker LD, Matson PA (1993) Nutrient limitations to plant growth during primary succession in Hawaii Volcanoes National Park Biogeochem 23: 197-215. https://doi.org/10.1007/BF00023752

Zhang CH (2014) Effects of grazing and fertilization on community productivity and species richness in eastern alpine meadow of Tibetan plateau. Pratacult Sci 31: 2293-2300. https://doi.org/10.11829\j.issn.1001-

0629.2014-0392. (in Chinese with English abstract)

Zhang T, Yang SB, Guo R, Guo JX (2016a) Warming and Nitrogen Addition Alter Photosynthetic Pigments, Sugars and Nutrients in a Temperate Meadow Ecosystem. Plos One 11. https://doi.org/10.1371/journal.pone.0155375

Zhang WJ, Zhang YQ, She WW, Qin SG, Feng W (2016b) Effects of Nitrogen Addition on Foliar Ecological Stoichiometric Characteristics of *Artemisia ordosica* Community. Res Environ Sci 29: 52-58. https://doi.org/10.13198/j.issn.1001-6929.2016.01.07. (in Chinese with English abstract)

Zhou XL, Guo Z, Zhang PF, Li HL, Chu CJ, Li XL, Du GZ (2017) Different categories of biodiversity explain productivity variation after fertilization in a Tibetan alpine meadow community. Ecol Evol 7: 3464-3474. https://doi.org/10.1002/ece3.2723

Source 3: References for the 13 experimental cases used to assess the response of species composition to N addition on the Tibetan Plateau.

Li J, Zhang C, Yang Z, Guo H, Zhou X, Du G (2017) Grazing and fertilization influence plant species richness via direct and indirect pathways in an alpine meadow of the eastern Tibetan Plateau. Grass Forage Sci 72: 343-354. https://doi.org/10.1111/gfs.12232

Qiu B, Luo YJ (2004) Effects of fertilizer gradients on productivity and species diversity in a degraded alpine meadow. J Lanzhou Univ Nat Sci 40: 56-59. https://doi.org/10.13885/j.issn.0455-2059.2004.03.014. (in Chinese with English abstract)

Shen H, Dong SK, Li SX, Yu Dan, Han YH, Zhang J (2019) Effects of nitrogen addition on the quantitative characteristics and photosynthesis of different plant functional groups in an alpine meadow of Qinghai-Tibetan Plateau. Chinese J Ecol 38: 1276-1284. https://doi.org/10.13292/j.1000-4890.201905.009. (in Chinese with English abstract)

Song MH, Yu FH, Ouyang H, Cao GM, Xu XL, Cornelissen JHC (2012) Different inter-annual responses to availability and form of nitrogen explain species coexistence in an alpine meadow community after release from grazing. Glob Change Biol 18: 3100-3111. https://doi.org/10.1111/j.1365-2486.2012.02738.x

Sun XM, Yu KL, Shugart HM, Wang G (2015) Species richness loss after nutrient addition as affected by N:C ratios and phytohormone GA_3 contents in an alpine meadow community. J Plant Ecol 9: 201-211. https://doi.org/10.1093/jpe/rtv037

Xu DH, Fang XW, Zhang RY, Gao TP, Bu HY, Du GZ (2015) Influences of nitrogen, phosphorus and silicon addition on plant productivity and species richness in an alpine meadow. AoB Plants 7: plv125. https://doi.org/10.1093/aobpla/plv125

Zhang CH (2014) Effects of grazing and fertilization on community productivity and species richness in eastern alpine meadow of Tibetan plateau. Pratacult Sci 31: 2293-2300. https://doi.org/10.11829\j.issn.1001-0629.2014-0392. (in Chinese with English abstract)

Zong N, Shi PL, Song MH, Zhang XZ, Jiang J, Chai X (2016) Nitrogen critical loads for an alpine meadow ecosystem on the Tibetan Plateau. Environ Manage 57: 531-542. https://doi.org/10.1007/s00267-015-0626-6

Zong N, Zhao GS, Shi PL (2019) Different sensitivity and threshold in response to nitrogen addition in four alpine grasslands along a precipitation transect on the northern Tibetan Plateau. Ecol Evol 9: 9782-9793. https://doi.org/10.1002/ece3.5514
